# Pheno-Ranker: a toolkit for comparison of phenotypic data stored in GA4GH standards and beyond

**DOI:** 10.1186/s12859-024-05993-2

**Published:** 2024-12-04

**Authors:** Ivo C. Leist, María Rivas-Torrubia, Marta E. Alarcón-Riquelme, Guillermo Barturen, PRECISESADS Clinical Consortium, Ivo G. Gut, Manuel Rueda

**Affiliations:** 1 Centro Nacional de Análisis Genómico, C/Baldiri Reixac 4, 08028 Barcelona, Spain; 2 Universitat de Barcelona (UB), Barcelona, Spain; 3 Pfizer–University of Granada–Junta de Andalucía Centre for Genomics and Oncological Research, Granada, Spain; 4 Institute of Environmental Medicine, Karolinska Institute, Stockholm, Sweden; 5 Department of Genetics, Faculty of Science, University of Granada, 18071 Granada, Spain; 6 Bioinformatics Laboratory, Centro de Investigación Biomédica, Biotechnology Institute, PTS, Avda del Conocimiento S/N, 18100 Granada, Spain

**Keywords:** GA4GH, Phenopacket v2, Beacon v2, Health data model, Semantic similarity, Genomics

## Abstract

**Background:**

Phenotypic data comparison is essential for disease association studies, patient stratification, and genotype–phenotype correlation analysis. To support these efforts, the Global Alliance for Genomics and Health (GA4GH) established Phenopackets v2 and Beacon v2 standards for storing, sharing, and discovering genomic and phenotypic data. These standards provide a consistent framework for organizing biological data, simplifying their transformation into computer-friendly formats. However, matching participants using GA4GH-based formats remains challenging, as current methods are not fully compatible, limiting their effectiveness.

**Results:**

Here, we introduce Pheno-Ranker, an open-source software toolkit for individual-level comparison of phenotypic data. As input, it accepts JSON/YAML data exchange formats from Beacon v2 and Phenopackets v2 data models, as well as any data structure encoded in JSON, YAML, or CSV formats. Internally, the hierarchical data structure is flattened to one dimension and then transformed through one-hot encoding. This allows for efficient pairwise (all-to-all) comparisons within cohorts or for matching of a patient’s profile in cohorts. Users have the flexibility to refine their comparisons by including or excluding terms, applying weights to variables, and obtaining statistical significance through Z-scores and *p*-values. The output consists of text files, which can be further analyzed using unsupervised learning techniques, such as clustering or multidimensional scaling (MDS), and with graph analytics. Pheno-Ranker’s performance has been validated with simulated and synthetic data, showing its accuracy, robustness, and efficiency across various health data scenarios. A real data use case from the PRECISESADS study highlights its practical utility in clinical research.

**Conclusions:**

Pheno-Ranker is a user-friendly, lightweight software for semantic similarity analysis of phenotypic data in Beacon v2 and Phenopackets v2 formats, extendable to other data types. It enables the comparison of a wide range of variables beyond HPO or OMIM terms while preserving full context. The software is designed as a command-line tool with additional utilities for CSV import, data simulation, summary statistics plotting, and QR code generation. For interactive analysis, it also includes a web-based user interface built with R Shiny. Links to the online documentation, including a Google Colab tutorial, and the tool’s source code are available on the project home page: https://github.com/CNAG-Biomedical-Informatics/pheno-ranker.

**Supplementary Information:**

The online version contains supplementary material available at 10.1186/s12859-024-05993-2.

## Background

Comparing phenotypic and clinical (“pheno-clinical”) data is essential in both research and clinical settings, facilitating informed decisions across a wide range of diseases [1, 2]. For instance, the use of similarity matching contributes to the accurate diagnosis of diseases by aligning patient profiles with comparable cases [3–5]. Similarity matching also has been used in the development of human disease networks, grouping diseases based on common traits to deepen our understanding of their origins [6]. These analytical methods are key in propelling medical research forward and improving patient care, offering essential insights into a variety of medical conditions.

Standardization of phenotypic data is crucial for enabling meaningful patient matching in clinical research [7]. Historically, researchers faced challenges in working with phenotypic data due to the lack of a unified format and consistent nomenclature across different research centers. This variation in data representation hindered data sharing, integration, and effective collaboration. To overcome these challenges, health data standards have been developed to promote the harmonization of variable names and values [8]. These standards provide a common framework for organizing and structuring data, allowing researchers to use standardized vocabularies to define the values for variables [9]. By using standardized terms, researchers can ensure that phenotypic data are described consistently, enabling more accurate and reliable similarity matching and analysis across different datasets and research centers [10, 11]. This standardization enhances data interoperability, facilitates data discovery, and promotes collaborative research efforts in the field of phenotypic analysis.

The use of standardized vocabularies has paved the way for performing matching based on terms (or semantic) similarity [12, 13]. In this regard, many methods have emerged to compare pheno-clinical data encoded with SNOMED CT [14], OMIM [15], ICD-10 [16] or HPO (Human Phenotype Ontology) [17] terms [4, 10, 18–36]. The widespread use of HPO in comparison methods is not coincidental, stemming from its foundation as a standardized vocabulary that structures data into a directed acyclic graph (DAG). This design allows each term to have a unique identifier and a set of relationships with other terms, facilitating the detailed representation of phenotypic abnormalities in human diseases and the hierarchical nature of disease terms and their interconnections. While HPO matching provides valuable insights, it is limited to phenotypic features. As a result, researchers often include genes, proteins or variants to expand the scope of their search [5, 37–49].

The GA4GH [50] has recently approved the Phenopacket v2 Schema [51], a standard for sharing and interoperability of pheno-clinical and genomic data, and Beacon v2 [52], which not only includes models for biological data but also provides an API specification for federated data discovery. Both standards are gaining popularity among research centers, for their structured and compact data schemas, which promote effective data sharing and integration initiatives [50]. A peculiarity of Beacon v2 and Phenopacket v2 schemas is that they do not strictly prescribe ontologies. Instead, they recommend certain ontologies for use within them [53]. For example, NCIt [54] or HPO terms can be used for describing phenotypic features, and diseases can be described using OMIM or ICD-10 terminologies. Currently, to the best of our knowledge, no tool is available that can directly perform comparisons of individuals in deeply nested data such as those present in Phenopackets v2 and Beacon v2 data models.

Here, we present Pheno-Ranker, an open-source toolkit designed for semantic similarity analysis of pheno-clinical data. It specifically supports GA4GH standards Phenopackets v2 and Beacon v2, accepting their JSON/YAML data exchange formats as input. This allows Pheno-Ranker to compare individuals within cohorts or to find the best matches of a patient profile in cohorts. While it primarily processes health-related data, Pheno-Ranker accepts as input any data serialized in JSON, YAML, and CSV formats. Researchers can include or exclude terms and define their own weightings for variables, empowering them to focus on relevant traits and prioritize specific comparisons. The output from Pheno-Ranker is text files that can be further analyzed (with scripts included in the tool’s GitHub repository [55] and the online documentation [56]) using unsupervised learning techniques such as clustering [57] and multidimensional scaling (MDS) [58], or through graph analytics [59–61]. This flexibility makes Pheno-Ranker a powerful tool not only in healthcare contexts but in any domain requiring data comparison. The software includes comprehensive online documentation (including a Google Colab notebook) and a web user interface built as an R Shiny app [62].

The remainder of this manuscript is structured as follows: the ‘Implementation’ section explains the components, data formats, and the algorithm of Pheno-Ranker. The ‘Results and Discussion’ section evaluates Pheno-Ranker’s performance on simulated and real-world datasets, and discusses its utility and limitations. The ‘Conclusion’ summarizes the key findings and potential future applications.

## Implementation

### Components

The core of Pheno-Ranker is a Perl module designed to be executed via a command-line interface (CLI) script (see online documentation [56]). In addition to the CLI, we developed a Web App User Interface (UI) based on R Shiny [63] for interactive analyses, available at https://pheno-ranker.cnag.eu (see full architecture in Additional file 1: Fig. SF1). During the development of the software, we encountered several critical issues, which are addressed in Additional file 2: Tab. ST1. Installation instructions for both the web application and CLI are provided in the ‘Download and Installation’ section of the article, as well as in the online documentation.

### Input files

Pheno-Ranker natively processes JSON and YAML text files. The two formats supported out-of-the-box are:

i. The *individuals* entity of Beacon v2 Models, encompassing the (top) terms *diseases, ethnicity, exposures, geographicOrigin, id, interventionsOrProcedures, karyotipicSex, measures, pedigrees, phenotypicFeatures, sex* and *treatments*, serialized into the data exchange file known as Beacon Friendly Format (BFF) [64, 65].
ii. The *phenopacket* top-element of the Phenopacket v2 schema that encompasses the terms *id*, *subject*, *phenotypicFeatures*, *measurements*, *biosamples*, *interpretations*, *diseases*, *medicalActions*, *files* and *metaData*, serialized into Phenotype Exchange Format (PXF) files [66].

Both file formats have a similar data structure in which biomedical data are hierarchically structured (i.e., tree-like) under the terms. Besides these formats, the software accepts any other JSON/YAML data, regardless of being biomedically related or not (movies, books, etc.) if they are accompanied by a configuration file. The documentation [56] has examples of how to use Pheno-Ranker with various types of data.

### Algorithm

The Pheno-Ranker computational algorithm works as follows (see also Additional file 1: Fig. SF2a):

1. Each object containing one individual (loaded from PXF or BFF files) for the reference cohort(s) is “flattened” into a one-dimensional hash data structure (i.e., associative array or lookup table) and the variables are initialized with weights of one. For terms that consist of arrays of objects (e.g., *phenotypicFeatures*), the element indices are replaced with the CURIE-style identifiers (“id”) from the *required* ontology class (see Additional file 1: Fig. SF2b). We used an ad-hoc filtering (that can be changed with a configuration file) to filter out variables that do not provide any value to the similarity.
2. We generate a global hash for the reference cohort(s) by utilizing the unique variables. The size of the hash depends on the number of variables present in the cohort. The algorithm is optimized to handle many variables, even exceeding 10,000 (e.g., when considering genomic variation data). To address any potential limitations, the algorithm allows selecting a subset of N random variables from the total available (with the flag –max-number-var).
3. We use the global hash to convert categorical data into numerical form through one-hot encoding. For each individual in the reference cohort(s), we create a binary string (also referred in the text as “binary vector” or simply as “vector”) reflecting the variables in the global hash. The characters within this vector coincide with the global hash’s sorted keys, marking a ‘1’ for each variable present in an individual’s data and a ‘0’ for absent variables. The length of the vector corresponds to the number of keys in the global hash, ensuring a uniform representation of each individual’s data in line with the global hash’s size.
4. When working with a target patient’s data from a JSON file, it is flattened using the same method as described in step one. We then calculate the patient’s binary vector using the global hash derived from the cohort, omitting any variables unique to the patient. This approach of excluding patient-specific variables makes it easier to search within unrelated databases that contain pre-computed data.
5. Compute metrics using the binary strings.

The software allows the user to include or exclude specific terms to make the comparison more precise. For instance, users can include terms like *sex, ethnicity*, and *measures* or exclude terms such as *id* from the comparison. The full set of terms can be found in the online documentation. Finally, if the files contain HPO terms, it is possible to include ascendant terms in the comparisons.

### Metrics and output

Pheno-Ranker operates in two modes: *cohort* mode and *patient* mode, with the metrics depending on the mode selected.

In *cohort mode*, Pheno-Ranker computes pairwise comparisons between individuals within the cohort(s). The default metric is the Hamming distance [67] which assesses dissimilarity by counting the differing positions between two binary strings (a distance of 0 indicates identical variables between two individuals). Additionally, the software can calculate Jaccard indexes [68] to measure similarity. The Jaccard index, more effective with incomplete data, measures the ratio of intersection over the union (a value of 1 indicates identical variables between two individuals). The resulting output is a symmetrical square matrix from all N x N comparisons (N = number of individuals). During development, we tested several other metrics and found minimal impact on the overall results (see Additional file 1: Fig. SF3). Additionally, since the binary vectors can be exported as text files, users have the flexibility to conduct further analysis using alternative metrics or tools if desired.

In *patient* mode, the goal is to compare patient data with all individuals in the reference cohort(s) through pairwise comparisons. By default, the output is a table listing the most similar individuals, sorted by Hamming distance, but the sorting method can be configured to use the Jaccard index instead. In this mode, assessing the statistical significance of match results is crucial as it enables users to make well-informed decisions. To facilitate this, we provide standardized values for both metrics in the form of Z-scores, along with their corresponding *p*-values (see extended information at Additional file 3).

All intermediate files generated by Pheno-Ranker (including associative arrays, and data on variable coverage in the cohort(s) can be exported using the –export option. In patient mode, the option –align exports “alignment” files. The software enables users to assign specific weights to any variable in the vector, with the weights adding repetitions to the characters in the binary digit string for efficient comparison without affecting computational speed (e.g., weight of 5 will create a string of ‘11111’ for that variable if present and ‘00000’ if absent) [69]. With regards to execution time, Additional file 1: Fig. SF9 provides detailed information for datasets of varying sizes.

### Included utilities

Pheno-Ranker comes with four additional utilities: (i) *bff-pxf-plot*, a script to create images with summary statistics from BFF/PXF files (see Additional file 1: Fig. SF7), (ii) *bff-pxf-simulator*, a script that generates simulated BFF/PXF files using randomized terms, (iii) *csv2pheno-ranker*, a script for converting any CSV file into a format compatible with Pheno-Ranker, facilitating the use of publicly available datasets (such as those found at Kaggle [70]), and (vi) scripts for encoding and decoding Pheno-Ranker data into QR (Quick Response) codes [71]. Here, the content for the code is the binary vector (see Implementation/Algorithm section and Additional file 1: Fig. SF8). As an estimate, the capacity for version 40-L QR code is 2,953 bytes, which theoretically enables the storage of up to 7,089 variables (uncompressed) per individual [72]. More information on these utilities can be found in Additional file 2: Table ST2 and the online documentation [56].

### Datasets and experimental setup

To assess the precision and efficiency of Pheno-Ranker, we used three datasets from different sources:

I. *Simulated datasets*: To generate the simulated data, we used the included utility *bff-pxf-simulator.* We tested Pheno-Ranker with both Beacon v2 Models and Phenopackets v2 with their respective data exchange formats (BFF and PXF). For clarity, we will show results using BFF, though the findings are applicable to PXF as well.
II. *Synthetic dataset*: The data are part of the ‘CINECA_synthetic_cohort_EUROPE_UK1’, which comprises 2,504 samples with genetic data derived from the 1000 Genomes Project’s phase 3 and the Geuvadis study [73]. Accompanying these genetic data are 76 synthetic phenotypic attributes (many of them incomplete) available through the UK Biobank [74]. The phenotypic data were “augmented” with ontology terms and structured as the *individuals* entity of the Beacon v2 Models. Finally, the data were serialized into BFF as described elsewhere [65]. The location of the file is provided in the ‘Availability of Data and Materials’ section.
III. *Use Case—PRECISESADS dataset*: As part of the 3TR (Taxonomy, Treatment, Targets, and Remission) project, funded by the European Commission through the Innovative Medicines Initiative, we obtained access to clinical data generated by the PRECISESADS Clinical Consortium (Additional file 4: Part A). The PRECISESADS cohort comprises 617 healthy controls and patients with seven systemic autoimmune diseases (SADs), including 99 cases of mixed connective tissue disease (MCTD), 106 cases of primary antiphospholipid syndrome (PAPS), 385 cases of primary Sjögren’s syndrome (pSjS), 376 cases of rheumatoid arthritis (RA), 469 cases of systemic lupus erythematosus (SLE), 402 cases of systemic sclerosis (SSc), and 166 cases of undifferentiated connective tissue disease (UCTD). The raw clinical data for these projects were stored in CSV format and comprised 92 variables, encompassing comorbidities, treatments, phenotypic characteristics of the different diseases, and recruitment information. The CSV file was converted to a format suitable to Pheno-Ranker with the included utility *csv2pheno-ranker*.

On these datasets we employed the metrics explained on the section ‘Metrics and output’ to measure the (dis)similarity among individuals. In *cohort* mode, the resulting symmetrical square matrix is represented through heatmaps with clusters (see examples at Fig. 3) using R software [57]. To the distance matrices, we applied multidimensional scaling (MDS), a type of non-linear dimensionality reduction technique, to visualize the similarity levels among individual cases within the dataset [58, 75] (R scripts are available at [76]). In the MDS plots, the X and Y axes denote the two most significant dimensions that encapsulate most of the variation in the distances among individuals, thereby offering a spatial depiction of the pairwise dissimilarities (see examples at Fig. 5 and Additional file 1: Fig. SF5d). Regarding timing, creating the 2D distance matrices was notably efficient, with all tasks completing in under one second on a single core of an Intel i9 Workstation. Note that tables and figures for the simulated and synthetic datasets can be reproduced using the commands available in the share directory of the GitHub repository [76].

## Results and discussion

### User flowchart

**Fig. 1:**
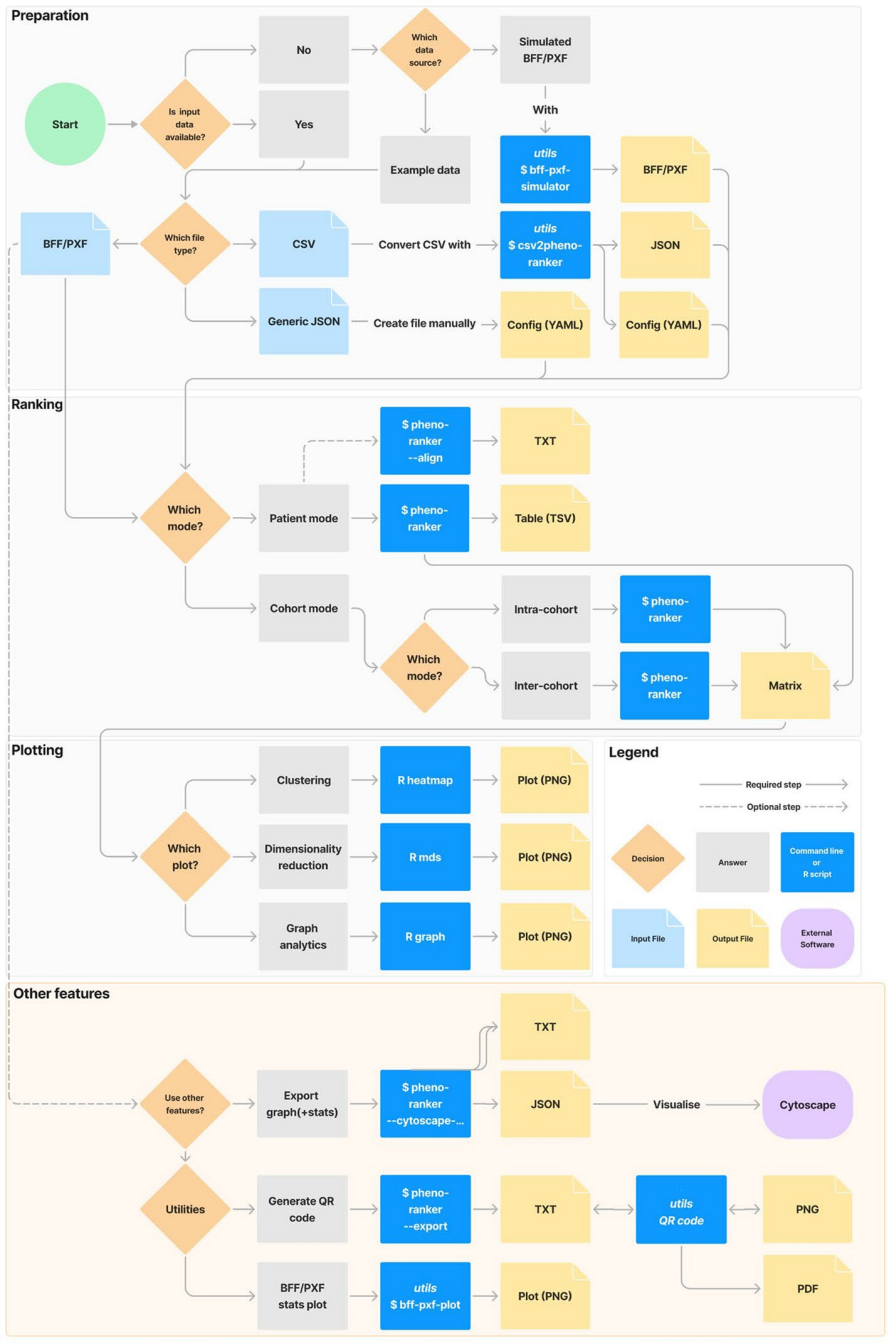
Flowchart of the available options in the Pheno-Ranker toolkit. ‘Preparation,’ ‘Ranking,’ and ‘Plotting’ can be performed using both the CLI and UI, while ‘Other Features’ are exclusive to the CLI. For more information, refer to the online documentation and Supporting Table 2

Figure 1 outlines a typical user workflow, divided into: (i) preparation, (ii) ranking, (iii) plotting, and (iv) other features. Steps (i) to (iii) are common to both the CLI and Web App UI, while (iv) utilizes the CLI utilities. Additional details, including versions, programming languages, documentation links, and brief descriptions, are provided in Additional file 2: Tab. ST2.

### User interface

**Fig. 2:**
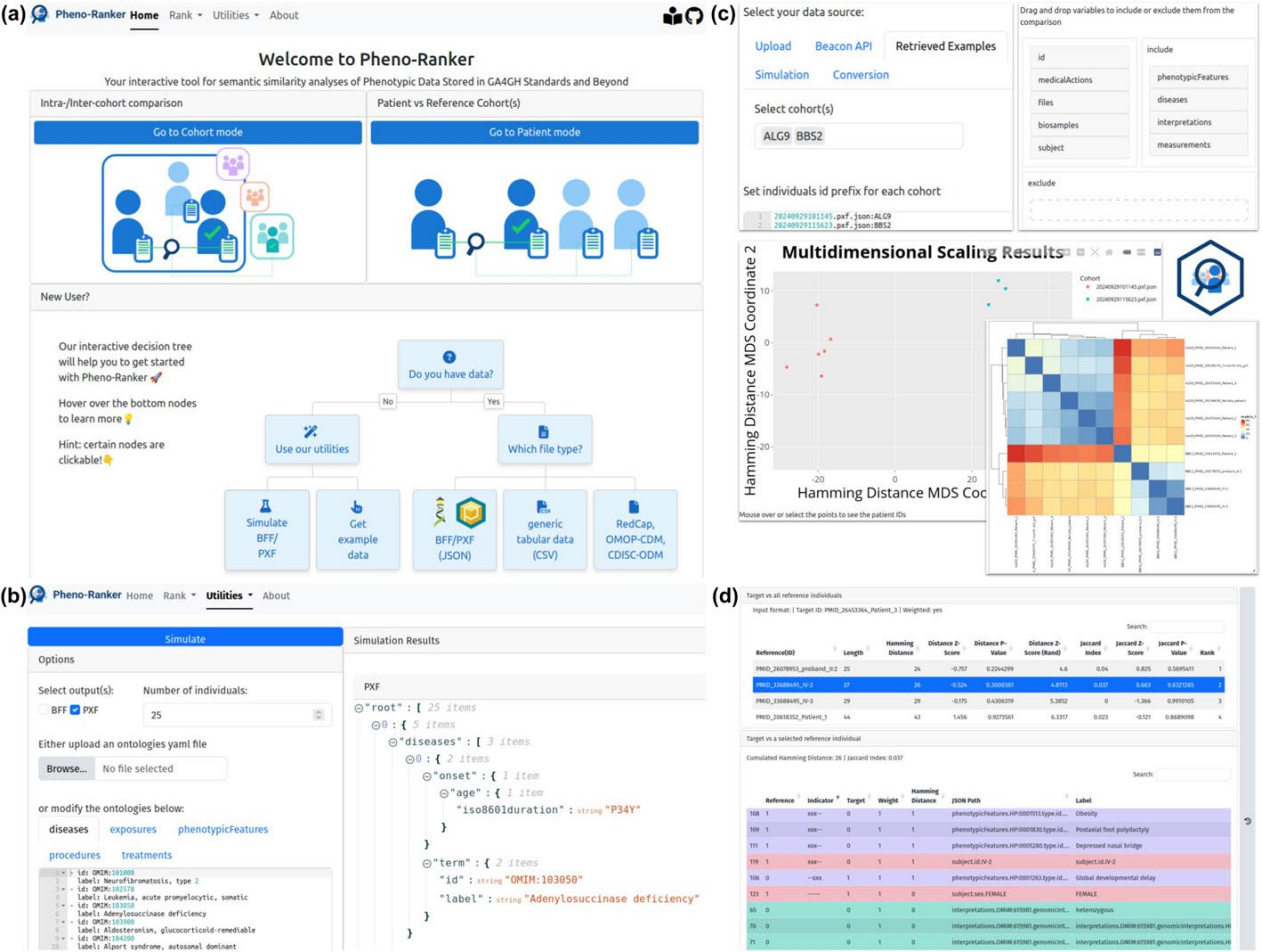
Screenshots of Pheno-Ranker User Interface. **a** Landing page, where returning users can access ranking modes, while new users can follow the interactive decision tree. **b** Utility for simulating synthetic patient data, customizable with user-defined ontologies. **c** *Cohort* mode settings and output: Individuals can be uploaded or retrieved from Pheno-Ranker-UI’s utilities. Top-level terms (e.g., *phenotypicFeatures*, *diseases*, *interpretations,…*) can be selected via drag-and-drop. Results are displayed as a heatmap or multidimensional scaling matrix scatter plot. These elements are also available in *patient* mode. **d** Tabular output of patient mode, showing differences between two individuals. The top table serves as a selector for the bottom table, which provides a one-to-one comparison of the target and reference patient (highlighted in blue). Rows are colored by top-level terms for clarity

We provide a web application (playground accessible at [62]) that offers a user-friendly interface, integrating Pheno-Ranker’s various command-line functionalities. A shared demo account is available for testing. For users working with sensitive data, secure access is provided through ORCID iD login. Returning users can quickly access the two ranking modes—cohort and patient—while new users can follow the interactive decision tree for guidance (see Fig. 2a).

Users can: (i) upload their own data in BFF, PXF, or CSV format leveraging the *csv2pheno-ranker* tool, which converts it into a Pheno-Ranker-compatible format, (ii) select simulated data obtained from the built-in *bff-pxf-simulator* (see Fig. 2b), (iii) use example data from a Phenopackets corpus [77] (see Fig. 2c), and, (iv) employ data directly queried from Beacon v2 APIs (record granularity) [52]. The UI output will vary depending on whether it is in cohort mode or in patient mode. In both modes, the software generates a variety of results such as heatmaps (with clusters) [78], dimensionality reduction plots [58, 75, 79] and graph-based plots [60, 80, 81] (see Fig. 2c). To the patient mode exclusive is the output of HTML tables (Fig. 2d) such as an alignment resembling that in BLAST [82]. A settings bar allows users to select datasets, define patient ID prefixes, and configure advanced options such as weighting variables and excluding specific terms via a drag-and-drop interface. Each utility and ranking mode includes a history sidebar for users to revisit, rename, or delete past runs, which are stored for at least 30 days.

### Simulated datasets

Our initial experiment aimed to validate Pheno-Ranker’s capability to handle the variability of information present in BFF files (JSON data structures). We created 10 and 100 completely random individuals by selecting 10 *diseases*, *exposures, phenotypicFeatures*, *procedures* and *treatments* (shuffled from pools of 100). Apart from the previous 50 properties, each individual was randomly assigned a biological *sex* (male or female) and a *ethnicity.* As shown in Additional File 1: Fig. SF5a and SF5c, which depict heatmaps of pairwise Hamming distances between all individuals in the cohort, the diagonal elements represent perfect matches (self-matches). The non-diagonal elements show distances ranging from 82 to 104, with a mean of 93.9 and a standard deviation of 4.3 for 10 individuals (see Additional File 1: Fig. SF4b), and from 74 to 106, with a mean of 94.2 and a standard deviation of 4.3 for 100 individuals (see Additional File 1: Fig. SF4d). These values are representative of a lower bound similarity (or similarity for a random model) using these number of variables. We used 10 and 100 individuals to show that the behavior is consistent, regardless of the number of individuals.

**Fig. 3:**
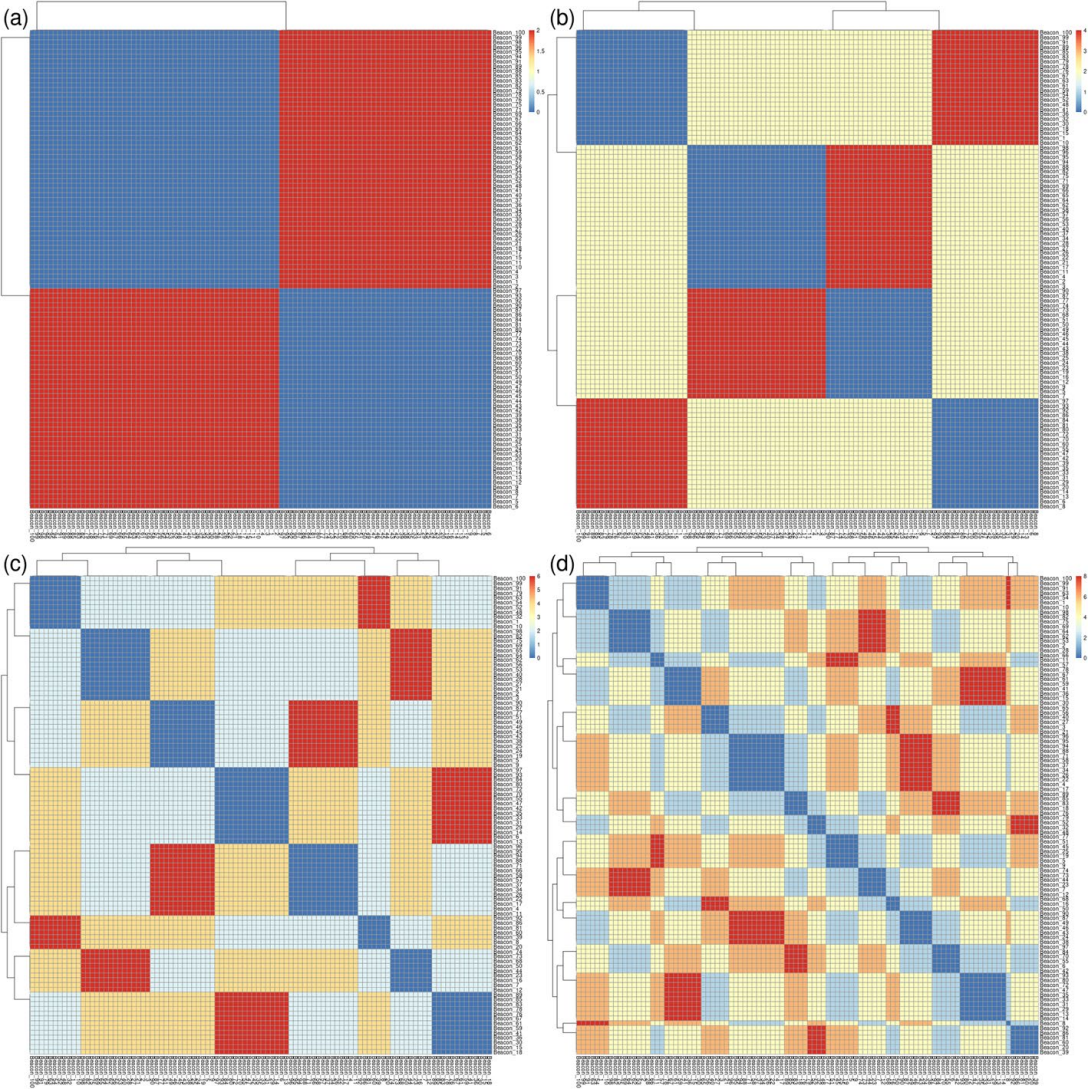
Heatmaps and clusters generated using Pheno-Ranker from simulated Beacon v2 data (*individuals* entity), based on pairwise Hamming distances between individuals. The dataset comprises 100 individuals, each with 1 *phenotypicFeatures*, 1 *disease*, and 1 *treatment*, randomly selected from a pool of two, along with their *sex* and *ethnicity*. **a** The heatmap shows two clusters when only phenotypic features are considered, representing the two distinct features in the pool. **b** By including sex, the clusters increase to four, distributing the two phenotypic features across both sexes. **c** Adding diseases to the analysis results in eight clusters. **d** Finally, incorporating treatments leads to a total of 16 clusters. Figure results can be reproduced by using the commands at [76]

The second experiment was designed to show the power of Pheno-Ranker for recognizing patterns in a cohort. For this purpose, we created 100 individuals, but this time, each individual had exactly 1 *phenotypicFeatures*, 1 *diseases*, and 1 *treatments* that were randomly selected from pools of 2 (note that the features, diseases and treatments chosen are irrelevant, they just serve for demonstration purposes), along with *sex* and *ethnicity*. When we only included *phenotypicFeatures*, the heatmap depicted two clusters, consisting of the two features from the pool (see Fig. 3a). When we repeat the analysis but this time including *sex*, the 2 clusters became 4 as the two *phenotypicFeatures* were spread equally among the females and males (see Fig. 3b). Continuing with this, we then included *diseases* on the analysis, and we got 8 clusters (see Fig. 3c). Finally, we included the *treatments* and we obtained 16 clusters (see Fig. 3d).

The third experiment aimed to demonstrate the effectiveness of assigning weights to variables. We simulated a cohort of 100 individuals, each with 2 *phenotypicMeasures*, 2 *diseases*, and 2 *treatments*, chosen from pools of 5, along with *sex* and *ethnicity*. In Additional file 1: Fig. SF5a we display the results when only including *phenotypicFeatures*, *diseases* and *treatments*. In Additional file 1: Fig. SF5b we include a weights file to the calculation, giving the variable ‘Caucasian’ a weight of 10. In Additional file 1: Fig. SF5c we also added the variable ‘RxNorm:1,000,000’ (Tribenzor) with a weight of 5. Finally, in Additional file 1: Fig. SF5d we display multidimensional data scaling on data from Additional file 1: Fig. SF5c.

The fourth experiment aimed to assess the ability to stratify patients in situations where data are incomplete or missing, a common issue in real-world datasets. We created two small cohorts of 10 individuals each to ensure labels remained visible in the plots. The first cohort consisted of individuals with a single disease (disease A), while the second cohort included individuals with two diseases, selected from a pool of five (A, B, C, D, and E). This approach generated C(5,2) = 10 unique combinations of two diseases, with disease A pairing with B, C, D, or E to form combinations like AB, AC, AD, and AE. Repetitions of features were not allowed. For a sufficiently large cohort, 40% of individuals in the second cohort were expected to carry disease A. In our sample, only ‘Beacon_7’ (female) and ‘Beacon_10’ (male) from the second cohort had disease A. These two individuals clustered with the first cohort, as shown in Additional File 1: Fig. SF6a, where only *diseases* term was considered. As expected, when the term *sex* was included, the number of clusters doubled (Additional File 1: Fig. SF6b). Recalculating using the Jaccard similarity index yielded the same results (Additional File 1: Fig. SF6c and SF6d).

The previous results demonstrate Pheno-Ranker’s effectiveness in *cohort mode* for classification. Additionally, its *patient mode* allows for comparing a single patient’s data against one or more cohorts, providing various metrics to evaluate the significance of these matches. To evaluate the robustness, and continuing with simulated data, we created a patient consisting of exactly 2 *phenotypicFeatures* sampled from a pool of 25. The reference cohort, comprising 1000 individuals, was created similarly. This setup yields a match probability of ~ 3 individuals, calculated as 1000 / *C*(25,2). In Table 1 we display the results for the first 10 matches. In this experiment, when including only *phenotypicFeatures* in the calculation, our patient got an exact match (distance = 0) with 3 individuals from the simulated reference cohort (Beacon_154, Beacon_444 and Beacon_697). As expected, the distances ranged from 0 (perfect match) to 4 (differences on each position of the 4-character vector) with a mean of 3.85 and a standard deviation of 0.36. To build on this experiment, we generated a new patient and a reference cohort of 1000 individuals, each with 3 *phenotypicFeatures*, 3 *diseases*, and 3 *treatments* chosen from pools of 5. The results, shown in Table 2, revealed an exact match for the new patient with two individuals in the cohort (Beacon_121 and Beacon_49), sharing all variables (9 out of 15 possible), resulting in a Z-score of − 3.38. This run highlights the Z-score metric’s stringency, as matches with a distance equal to 2 (which still have 8 matches) were not deemed significant. It is important to note that the Z-score’s significance is expected to be more pronounced with real data, which typically does not have perfect matches.

**Table 1:** Descriptors from the comparison between a given simulated patient and a cohort of 1000 individuals with 2 *phenotypicFeatures* sampled from a pool of 25 features. The patient was created with the same conditions. Table results can be reproduced by using the commands at [76]

Reference(ID)	Length	Hamming-distance	Distance-*Z*-score	Distance-*P*-value	Distance-*Z*-score(rand)	Jaccard-index	Jaccard-*Z*-score	Jaccard-*P*-value
Beacon_154	2	0	− 5.093	0.0000002	− 1.4142	1.000	7.447	0.0000000
Beacon_444	2	0	− 5.093	0.0000002	− 1.4142	1.000	7.447	0.0000000
Beacon_697	2	0	− 5.093	0.0000002	− 1.4142	1.000	7.447	0.0000000
Beacon_385	3	2	− 2.343	0.0095640	0.5774	0.333	2.219	0.1113630
Beacon_253	3	2	− 2.343	0.0095640	0.5774	0.333	2.219	0.1113630
Beacon_192	3	2	− 2.343	0.0095640	0.5774	0.333	2.219	0.1113630
Beacon_977	3	2	− 2.343	0.0095640	0.5774	0.333	2.219	0.1113630
Beacon_975	3	2	− 2.343	0.0095640	0.5774	0.333	2.219	0.1113630
Beacon_898	3	2	− 2.343	0.0095640	0.5774	0.333	2.219	0.1113630
Beacon_886	3	2	− 2.343	0.0095640	0.5774	0.333	2.219	0.1113630

**Table 2:** Descriptors from the comparison between a given simulated patient and a cohort of 1000 individuals with 3 *phenotypicFeatures*, 3 *diseases* and 3 *treatments* from pools of 5. The patient was created with the same conditions. Table results can be reproduced by using the commands at [76]

Reference(ID)	Length	Hamming-distance	Distance-*Z*-score	Distance-*P*-value	Distance-Z-score(RAND)	Jaccard-index	Jaccard-*Z*-score	Jaccard-*P*-value
Beacon_121	9	0	− 3.381	0.0003608	− 3.0000	1.000	4.442	0.0002886
Beacon_49	9	0	− 3.381	0.0003608	− 3.0000	1.000	4.442	0.0002886
Beacon_821	10	2	− 2.433	0.0074969	− 1.8974	0.800	2.847	0.0323852
Beacon_56	10	2	− 2.433	0.0074969	− 1.8974	0.800	2.847	0.0323852
Beacon_666	10	2	− 2.433	0.0074969	− 1.8974	0.800	2.847	0.0323852
Beacon_74	10	2	− 2.433	0.0074969	− 1.8974	0.800	2.847	0.0323852
Beacon_829	10	2	− 2.433	0.0074969	− 1.8974	0.800	2.847	0.0323852
Beacon_343	10	2	− 2.433	0.0074969	− 1.8974	0.800	2.847	0.0323852
Beacon_337	10	2	− 2.433	0.0074969	− 1.8974	0.800	2.847	0.0323852
Beacon_365	10	2	− 2.433	0.0074969	− 1.8974	0.800	2.847	0.0323852

### Synthetic dataset

**Fig. 4:**
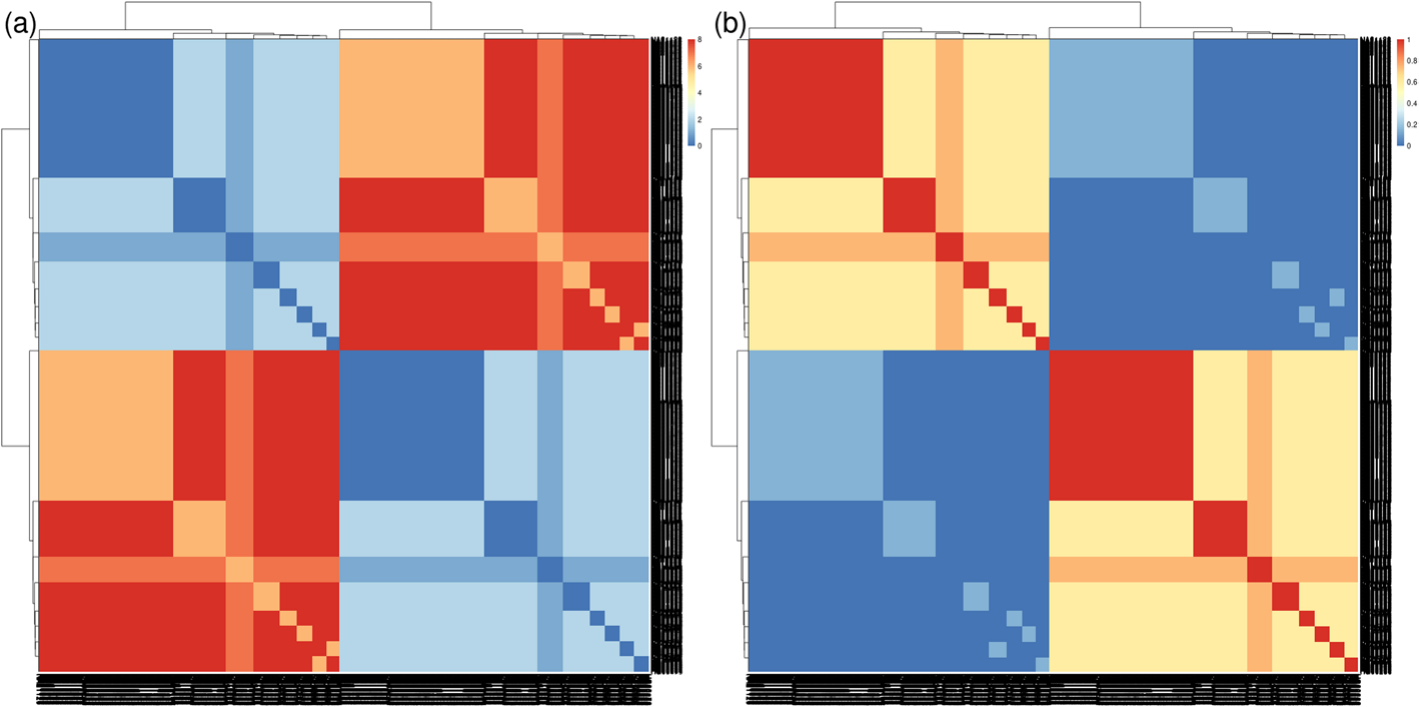
Heatmaps and clusters generated using Pheno-Ranker from synthetic Beacon v2 data (individuals entity), based on pairwise Hamming distances between individuals. The synthetic dataset ‘CINECA_synthetic_cohort_EUROPE_UK1’ includes 2504 individuals, with terms *sex* and *ethnicity* incorporated, and weights of 3 assigned to sex. **a** Displays the heatmap based on Hamming distance, reflecting dissimilarity among individuals. **b** Shows the heatmap according to the Jaccard index, highlighting similarities. Figure results can be reproduced by using the commands at [76]

Next, we used the synthetic data from the ‘CINECA_synthetic_cohort_EUROPE_UK1’ dataset (see Implementation section, Dataset II), which consists of 2504 individuals. The purpose of this test was to check whether Pheno-Ranker was able to handle a dataset that is regularly used to debug Beacon v2 API deployments [65]. We performed calculations in *cohort* mode, including the terms *sex* and *ethnicity* and we gave a weight of 3 to the term *sex* that included variables ‘sex.id.NCIT:C20197’ and ‘sex.id.NCIT:C16576’: In Fig. 4a we display the heatmap according to Hamming distance (dissimilarity) and Fig. 4b according to Jaccard index (similarity). Note that both metrics yield similar yet complementary results.

### Use case—PRECISESADS dataset

In the previous sections, Pheno-Ranker has undergone extensive testing with simulated and synthetic datasets. However, it is important to acknowledge that such datasets may not fully encompass the variability present in real-world data, as they often contain a consistent number of variables, unlike the more complex and variable data typically encountered in clinical trials. Therefore, we conducted an analysis on the PRECISESADS clinical records to demonstrate the utility of the tool with a real-world dataset. The analyses were performed by incorporating all available information from the PRECISESADS cohort, with non-informative values (e.g., unknown, missing) replaced by ‘NA’ (i.e., Not Available).

**Fig. 5:**
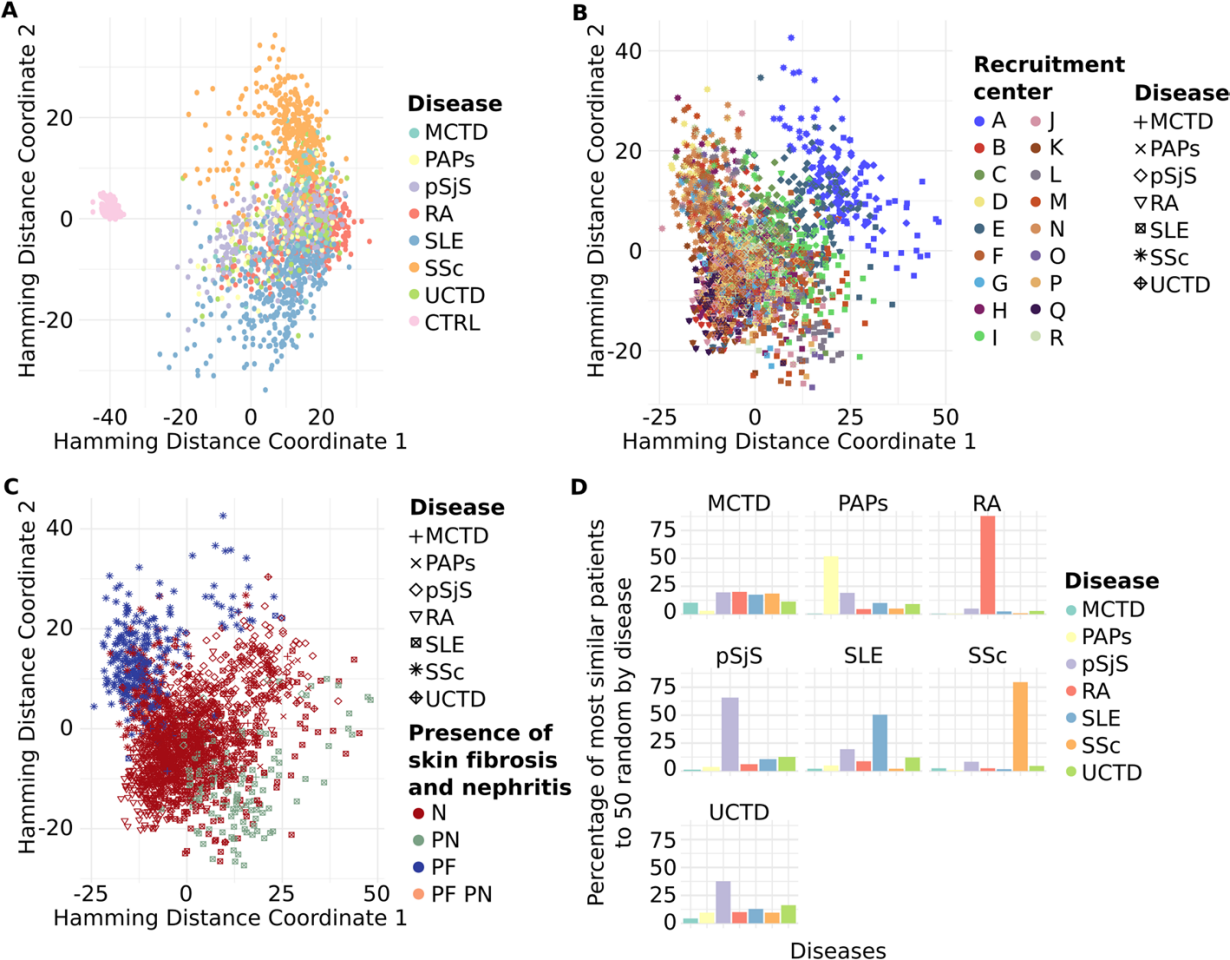
Pheno-Ranker’s analysis of the PRECISESADS dataset involves 7 disease-specific cohorts and 1 control cohort. Figures **a**–**c** showcase plots generated through multidimensional data scaling (MDS) applied to the distance matrix from an inter-cohort (cohort mode) analysis. Specifically, **a** demonstrates the MDS of the 8 cohorts, including the control, with each cohort colored differently; **b** presents the MDS plot of the 7 diseases, differentiated by research center and symbolized for each disease; and **c** shows the MDS plot of the 7 diseases, highlighted by the presence of skin fibrosis and nephritis. Figure **d** illustrates the Pheno-Ranker’s performance on patient mode, revealing the percentage of the most similar patients (from all diseases) to 50 randomly selected patients for each disease

With the *cohort* mode, we aimed to identify hidden potential confounders and/or clinical features that might differentiate between patients. As anticipated, most clinical features and comorbidities were absent in healthy controls, consistent with the definition of healthy control recruitment, resulting in a distinct cluster separate from the patients (Fig. 5a). SADs are known to share phenotypic commonalities, reflected in a single cluster, but with a more dispersed distribution than that of healthy controls. Notably, two diseases, SSc and SLE, stood out from the other five, distributed in the upper and lower parts of the SADs clusters (Fig. 5a).

To further investigate the characteristics distinguishing SADs patients, *cohort* mode was rerun excluding the healthy controls, and ANOVA analyses were conducted between MDS dimensions and each included variable (see Additional file 5 and R script file [76]). The primary variable associated with both MDS dimensions was the recruitment center. This was reflected in the position of center ‘A’ in the SADs cluster during dimensional reduction analysis (Fig. 5b). A detailed examination of the variables revealed that center ‘A’ exhibited the highest proportion of missing values (data not shown), potentially explaining the observed difference. Subsequently, the next two variables most strongly associated with the MDS dimensions were the presence of skin fibrosis, a hallmark of SSc [83], and the presence of nephritis, a major and quite specific clinical complication in SLE patients [84]. These findings elucidated the separation of these two diseases from the rest of the SADs (Fig. 5c).

Finally, we applied Pheno-Ranker in *patient* mode to evaluate its patient-matching capabilities. Figure 5d displays the percentage of patients most similar to 50 randomly chosen individuals from each disease group. Notably, Pheno-Ranker reliably identified similarities across various diseases. Yet, it faced difficulties with Mixed Connective Tissue Disease (MCTD) and Undifferentiated Connective Tissue Disease (UCTD) due to the shared phenotypic features with other conditions.

### Features, capabilities, and limitations

Coding schemes like HPO or OMIM terms have greatly enhanced genomic and phenotypic data analysis. Their use boosts semantic similarity analysis, making it easier to identify related terms and concepts, thus enriching our data understanding. To compare phenotypic data encoded in these schemes, various methods have emerged, including text-based comparisons and analyses of term relationships from ontology structures [4, 10, 18–36].

Here, we present Pheno-Ranker, an innovative approach that extends beyond the constraints of tools dependent on pre-selected ontologies. We want to clarify that Pheno-Ranker is not intended to outperform these types of tools. If a researcher wants to compare individuals based on HPO terms (or others such as OMIM, SNOMED CT, etc.), Pheno-Ranker’s only strength lies in its ability to directly process BFF or PXF data exchange formats, eliminating the need for data conversions. Rather, its uniqueness lies in the versatility and detail it offers; it is compatible with any standardized terminology leading to more nuanced and flexible outcomes. Pheno-Ranker handles data in JSON/YAML formats, first flattening the data to 1D lookup tables and then converting them into binary digit strings. This conversion conserves data context and streamlines efficient similarity matching, also ensuring compatibility with machine learning algorithms. To our knowledge, Pheno-Ranker is the first tool to directly perform similarity calculations on two GA4GH standards, making it particularly suitable for patient matching (matchmaking) and stratification in GA4GH-compliant data repositories. The software can be configured to work with other health data standards, like OpenEHR [85], as shown in the example at the online documentation [56]. Additionally, in conjunction with our Convert-Pheno tool [86], it supports data from other clinical data models such as the Observational Medical Outcomes Partnership Common Data Model (OMOP CDM), ensuring compatibility with various health data ecosystems.

Pheno-Ranker includes a suite of utilities, significantly broadening its use beyond YAML or JSON hierarchical data formats. Firstly, *bff-pxf-plot* is a tool developed to create summary statistics plots as PNG images for BFF and PXF formats (see Additional file 1: Fig. SF7 and online documentation [56]). Secondly, *bff-pxf-simulator* is designed to generate simulated data in BFF/PXF exchange formats, addressing the scarcity of synthetic or simulated datasets for Beacon v2 and Phenopackets v2 in the literature [87, 88]. Our simulated data are versatile, serving purposes ranging from technical, like testing Beacon v2 implementations [65], to scientific, such as creating “control” cases for outlier detection. Thirdly, the *csv2pheno-ranker* utility enables converting CSV files into a format compatible with Pheno-Ranker. This expands the scope of analysis, for example, enabling comparison of samples in a multi-sample VCF based on their “genomic fingerprint”, as shown in our documentation [56]. Fourthly, we offer a set of utilities for encoding/decoding the binary vector into QR codes (PNG images) plus generating PDF reports (see Additional file 1: Fig. SF8 and examples at the documentation [56]). This concept aligns with the health passports [89] that store patient information in barcodes (i.e., those used to encode in COVID-19 vaccination state). Pheno-Ranker efficiently condenses data by directly encoding binary vectors into QR codes, enhancing the capacity for storage of variables within the code. Although currently a proof of concept, our solution could be applied to health data transfer, especially with the implementation of proper security measures, such as data encryption [8]. Other futuristic applications could be patient enrolment in clinical trials or “wireless” similarity matching via a Pheno-Ranker mobile app.

The simplicity of our algorithm makes it suitable for personalized implementations, allowing users to store the one-hot encoded data in a database for efficient comparisons using for instance SQL functions or vector databases. The Pheno-Ranker algorithm could easily be implemented in a federated network, such as Beacon v2 networks (see explanation at Additional file 6). This approach holds promise for collaborative research, such as hospital networks, aiming to find similar patients across different institutions.

One limitation of Pheno-Ranker is its handling of continuous data, such as age or numerical measurements. For age data, both the Beacon v2 and Phenopackets v2 schemas allow the use of ranges instead of specific ages, which enhances privacy. For other continuous variables, we recommend using ranges or pre-processing them into bins/categories whenever possible. Cautious handling of continuous data is essential, depending on the research goals and the nature of the data.

Another limitation of our method is its lack of terminology-matching capability, meaning that variables describing identical concepts across different standardized vocabularies are not recognized as matches. This limitation arises not from a flaw in our approach, but rather from the lack of prescribed ontologies within the Beacon v2 and Phenopacket v2 schemas. Additionally, our method currently supports only exact matches, excluding the possibility of fuzzy searches. Despite these challenges, the rapidly advancing field of ontology mapping, coupled with the ongoing progress in natural language processing (NLP), holds promise for overcoming these limitations soon.

Pheno-Ranker has been rigorously tested using a variety of simulated and synthetic datasets. However, these datasets may not encompass the full variability found in real-world data, like that from clinical trials. To address this, we also analyzed data from the PRECISESADS project, which enabled us to stratify diseases by their unique characteristics, identify outliers, and match patients with the same disease across the entire dataset.

It is important to note, though, that in case–control comparisons, the number of variables between groups can skew similarity measures. The Hamming distance may be artificially higher for cases with more variables, requiring normalization or a focus on shared variables. The Jaccard index, while less impacted, can underestimate similarity in cases with many additional variables. Addressing this bias involves selecting key variables, considering data imputation for missing values, and performing sensitivity analyses. Expert input is vital for including/excluding relevant variables and validating results. Pheno-Ranker’s ability to tailor analysis to key traits demonstrates its capacity for delivering valuable insights from complex datasets.

Data security and privacy are crucial when deploying Pheno-Ranker. Each center is responsible for maintaining the confidentiality, integrity, and availability of their data, and must implement appropriate safeguards to protect sensitive information.

At CNAG, we are preparing to assess Pheno-Ranker’s effectiveness with PXF data from the Genome-Phenome Analysis Platform [90], and exploring its capabilities in federated analysis contexts (refer to the Additional file 6). In the broader scope of Pheno-Ranker’s capabilities, its applicability is not just confined to genomic and phenotypic data. It can be effectively adapted for use with diverse JSON or CSV datasets, provided an appropriate configuration file is used. This feature significantly widens the range of potential applications, enabling researchers to investigate semantic similarities in various fields.

In conclusion, Pheno-Ranker is a versatile and powerful tool for individual-level comparison of pheno-clinical data. By uncovering hidden patterns and relationships, Pheno-Ranker has the potential to advance personalized medicine, disease classification, and scientific discovery. We look forward to further developments and real-world applications of Pheno-Ranker, leveraging its capabilities to drive progress in genomics and beyond.

## Conclusions

We introduce Pheno-Ranker, an open-source software for phenotypic data analysis applicable in research and clinical settings. Compatible with Beacon v2 and Phenopackets v2 data models, it processes data in JSON, YAML, or CSV formats across any domain. Pheno-Ranker offers two operational modes: cohort comparison and individual matching to the closest profiles within cohorts. The output consists of structured text files, which can be further analyzed using included R scripts for unsupervised learning techniques such as clustering, multidimensional scaling and graph analytics, broadening their applicability across various analytical tasks.

Pheno-Ranker includes a user-friendly R Shiny web application and a command-line interface, catering to different user preferences. Multiple installation options, including a containerized version, ensure seamless integration into existing workflows, while online documentation and an interactive Google Colab notebook offer guidance. The software also comes equipped with a suite of utilities that enable generation of simulated data, data visualization through plotting, and creation of QR codes, enhancing its practical utility. Pheno-Ranker enhances research and clinical workflows by enabling comparison of phenotypic data, helping researchers identify significant patterns, match patients, construct cohorts, and explore genotype–phenotype relationships.

### Availability and requirements

- *Project name*: Pheno-Ranker
- *Project home page*: https://github.com/CNAG-Biomedical-Informatics/pheno-ranker
- *Operating system* (*s*): Linux, MacOS
- *Programming languages*: Perl, Python, R, JavaScript
- *Other requirements*: Docker
- *License*: Artistic License 2 (CLI) and GNU GPL v3 (UI)
- *Any restrictions to use by non-academics*: None

### Download and installation

Pheno-Ranker can be installed locally on Linux or macOS. For the CLI, we offer five installation options, including from GitHub source, containerized options (Docker), and CPAN, depending on whether all utilities are needed (see Additional file 2: Table ST2 and https://cnag-biomedical-informatics.github.io/pheno-ranker/download-and-installation). The R Shiny web application can be easily deployed using Docker for a streamlined setup.

## Supplementary Information

Additional file1 (PDF 3430 KB)

Additional file2 (PDF 142 KB)

Additional file3 (PDF 62 KB)

Additional file4 (PDF 145 KB)

Additional file5 (XLSX 32 KB)

Additional file6 (PDF 224 KB)

## Data Availability

All simulated data presented in this paper can be replicated using the utility *bff-pxf-simulator*, which is accessible via CPAN distribution, containerized versions, GitHub repository at https://github.com/CNAG-Biomedical-Informatics/pheno-ranker/tree/main/utils/bff_pxf_simulator, and through the web app user interface (https://pheno-ranker.cnag.eu). Synthetic data for the CINECA cohort is available at https://github.com/EGA-archive/beacon2-ri-tools/blob/main/CINECA_synthetic_cohort_EUROPE_UK1/bff. PRECISESADS clinical dataset is hosted by ELIXIR Luxembourg (URL: https://elixir-luxembourg.org). The dataset is available upon request, and the access procedure is described on the data landing page (doi.org/10.17881/th9v-xt85).
