# Supplementary material for: Pheno-Ranker: a toolkit for comparison of phenotypic data stored in GA4GH standards and beyond
Source: BMC Bioinformatics. 2024 Dec 4;25:373. doi: 10.1186/s12859-024-05993-2 (PMC11616229; doi:10.1186/s12859-024-05993-2)
Supplement: Supplementary file 1 — Additional file1 (PDF 3430 KB) [file 12859_2024_5993_MOESM1_ESM.pdf]

Supporting Figure 1

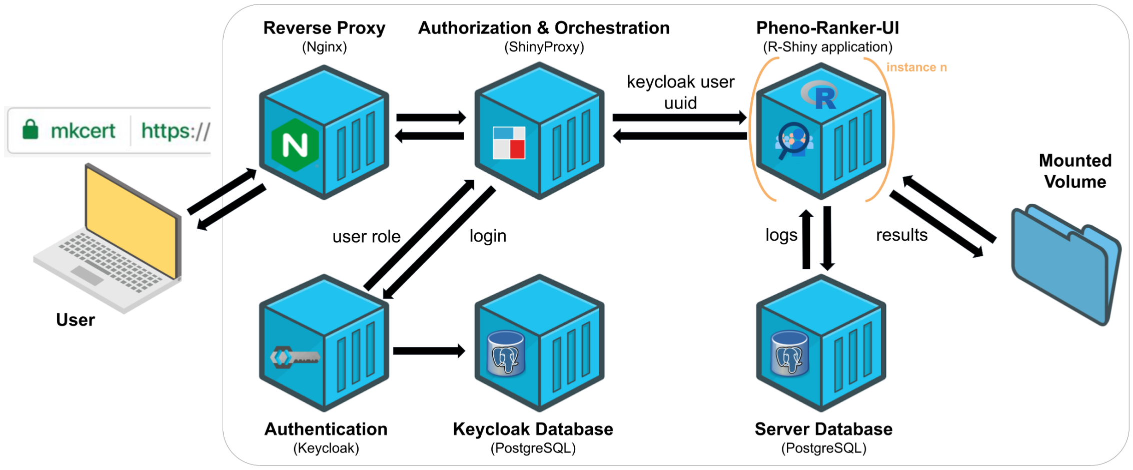

Figure SF1. Pheno-Ranker Web App UI architecture. Diagram of the architecture for Pheno-Ranker's Web App UI, which uses microservices and a *nginx* reverse proxy. *Shinyproxy* is used for authorization and orchestration of the Pheno-Ranker instances. *Keycloak* is used for user authentication and management. Each started instance is mounted to a shared volume and connected to a database, allowing for file access and storage of the Pheno-Ranker results and retrieval of past runs.

Supporting Figure 2

(a)

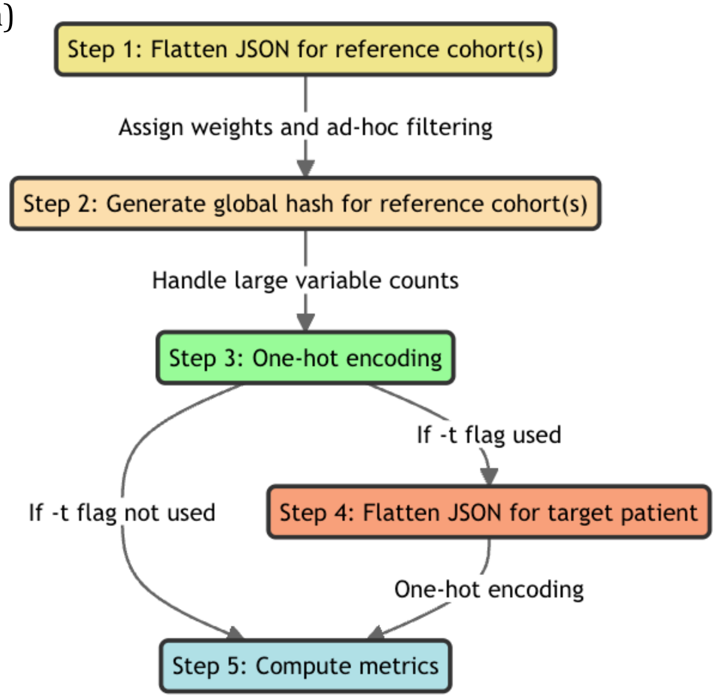

(b)

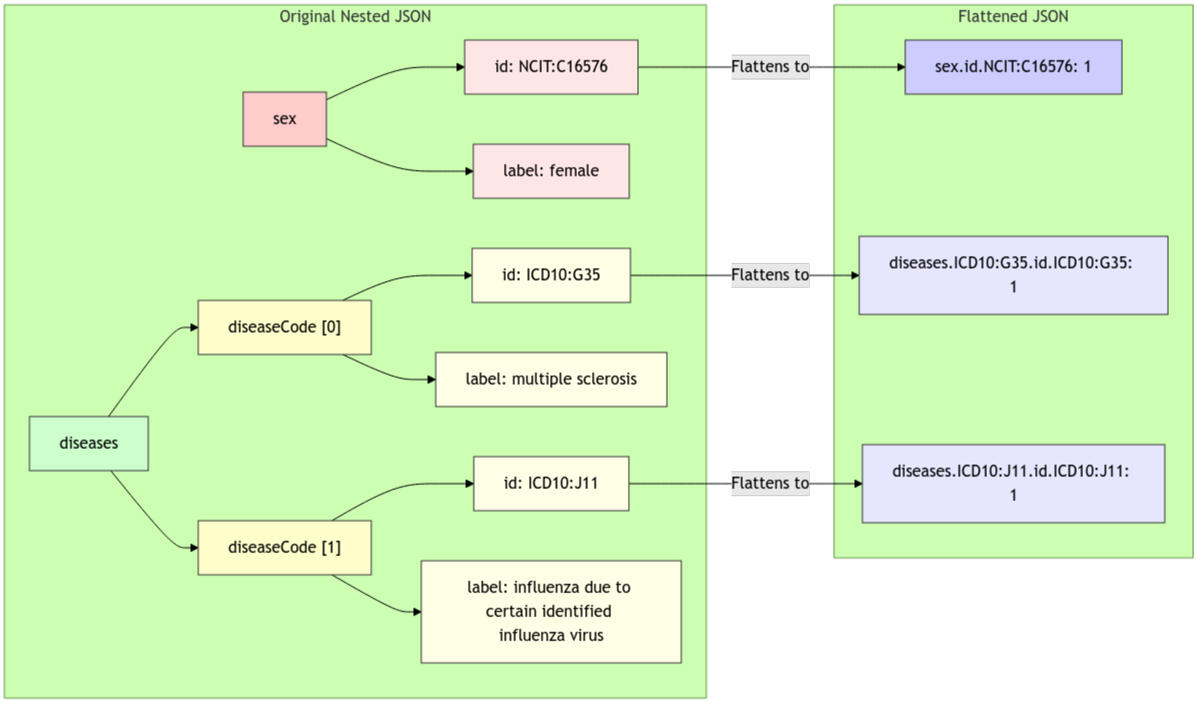

Figure SF2. Overview of the Pheno-Ranker algorithm. (a) Schematic overview of the Pheno-Ranker algorithm, and (b) detailed view of Step 1 – transformation from a nested

to a flattened JSON structure. During the flattening process, the ‘label’ fields are excluded using a regular expression specified by the ‘exclude_properties_regex’ parameter in the configuration file. The flattened keys preserve the original hierarchical relationships within the data.

Supporting Figure 3

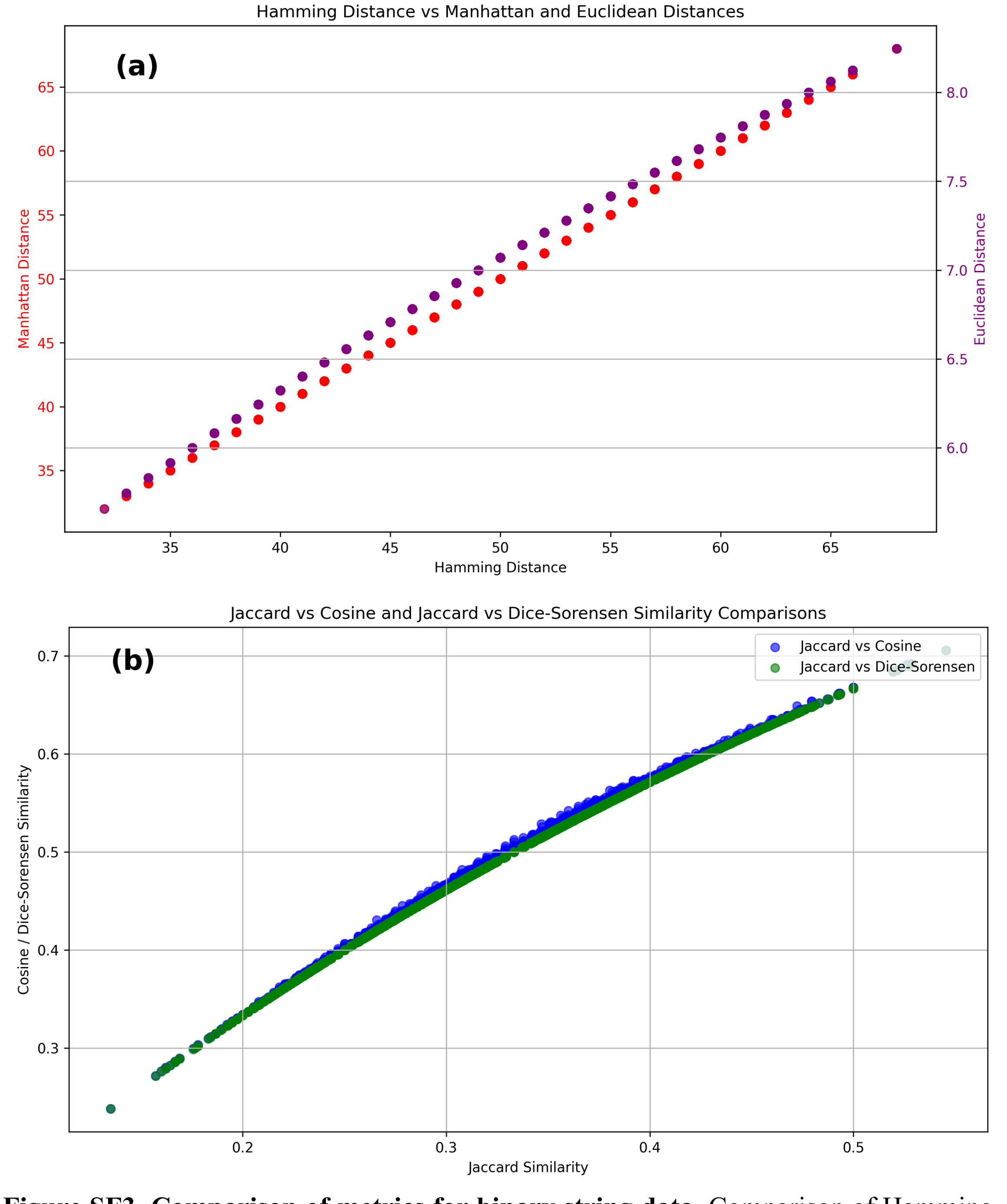

Figure SF3. Comparison of metrics for binary string data. Comparison of Hamming-based dissimilarity and Jaccard-based similarity metrics for 100 randomly generated binary strings, each 100 characters long. **(**a) shows the relationship between Hamming, Manhattan, and Euclidean distances, highlighting the effect of different distance-based

metrics. (b) compares Jaccard similarity with Cosine and Dice-Sorensen similarities. The results show that the choice of metric—whether distance-based (Hamming) or similarity-based (Jaccard, Cosine, Dice-Sorensen)—does not qualitatively affect the outcomes. We used Hamming and Jaccard because they are intuitive and widely adopted, but the results indicate that other metrics would lead to similar conclusions.

Supporting Figure 4

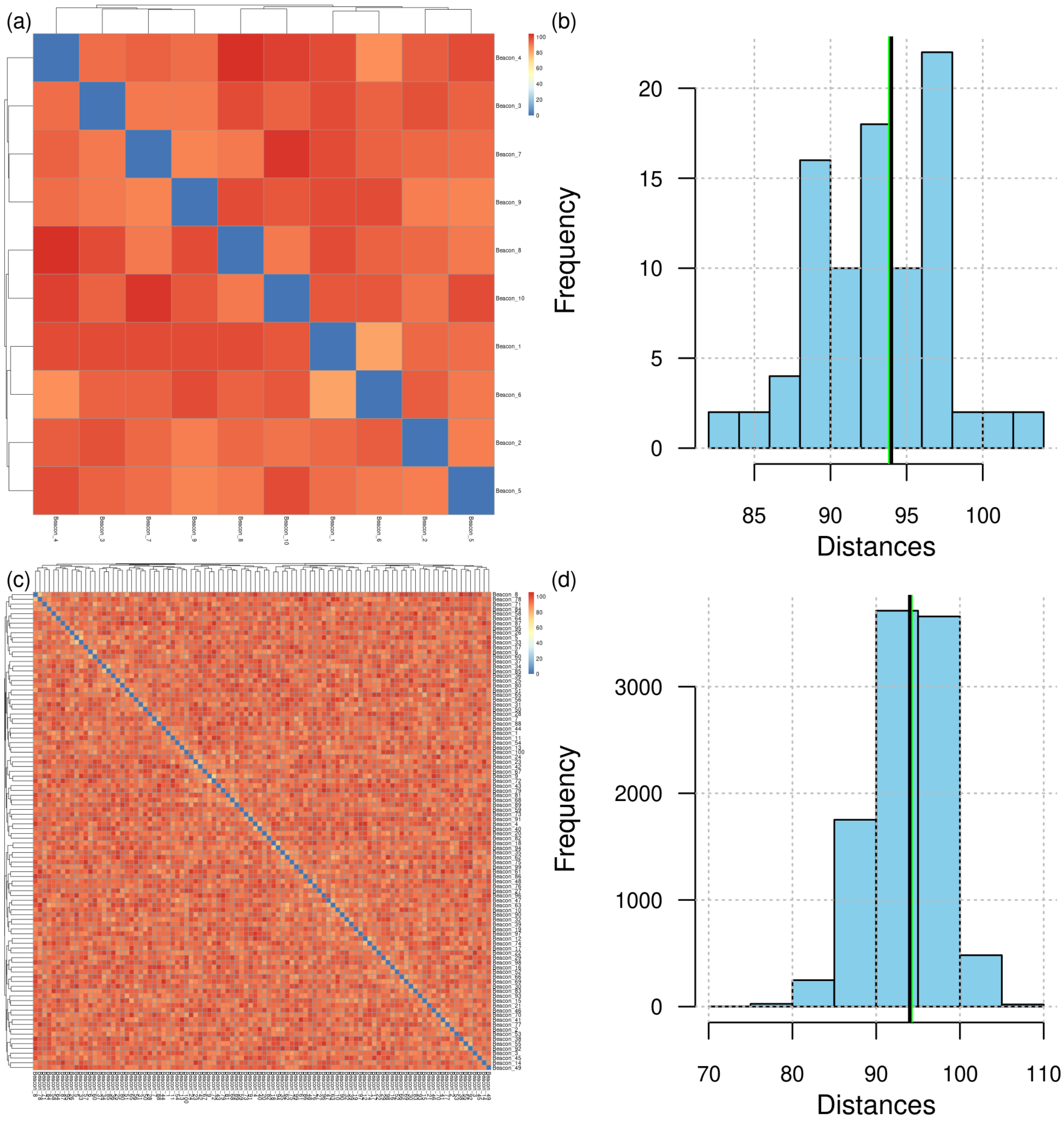

Figure SF4. Results from datasets of randomly simulated data. Results from simulated data from the *individuals* entity of the Beacon v2 Models using Pheno-Ranker, created from the pairwise Hamming distance between all individuals. Heatmaps and clusters obtained from: (a) 10 individuals with 10 *phenotypicFeatures*, 10 *diseases*, and 10 *treatments*, 10 *exposures* and 10 *interventionsOrProcedures*, plus *id*, *sex* and *ethnicity*.

(c) 100 individuals with the same properties described in (a). (c) Histogram distribution of distances for (a). (d) Histogram of distances for (c). As a reference, the mean is drawn as a green line and the median as a black line. Figure results can be reproduced by using the commands at https://github.com/CNAG-Biomedical-Informatics/pheno-ranker/tree/main/share/fig.

Supporting Figure 5

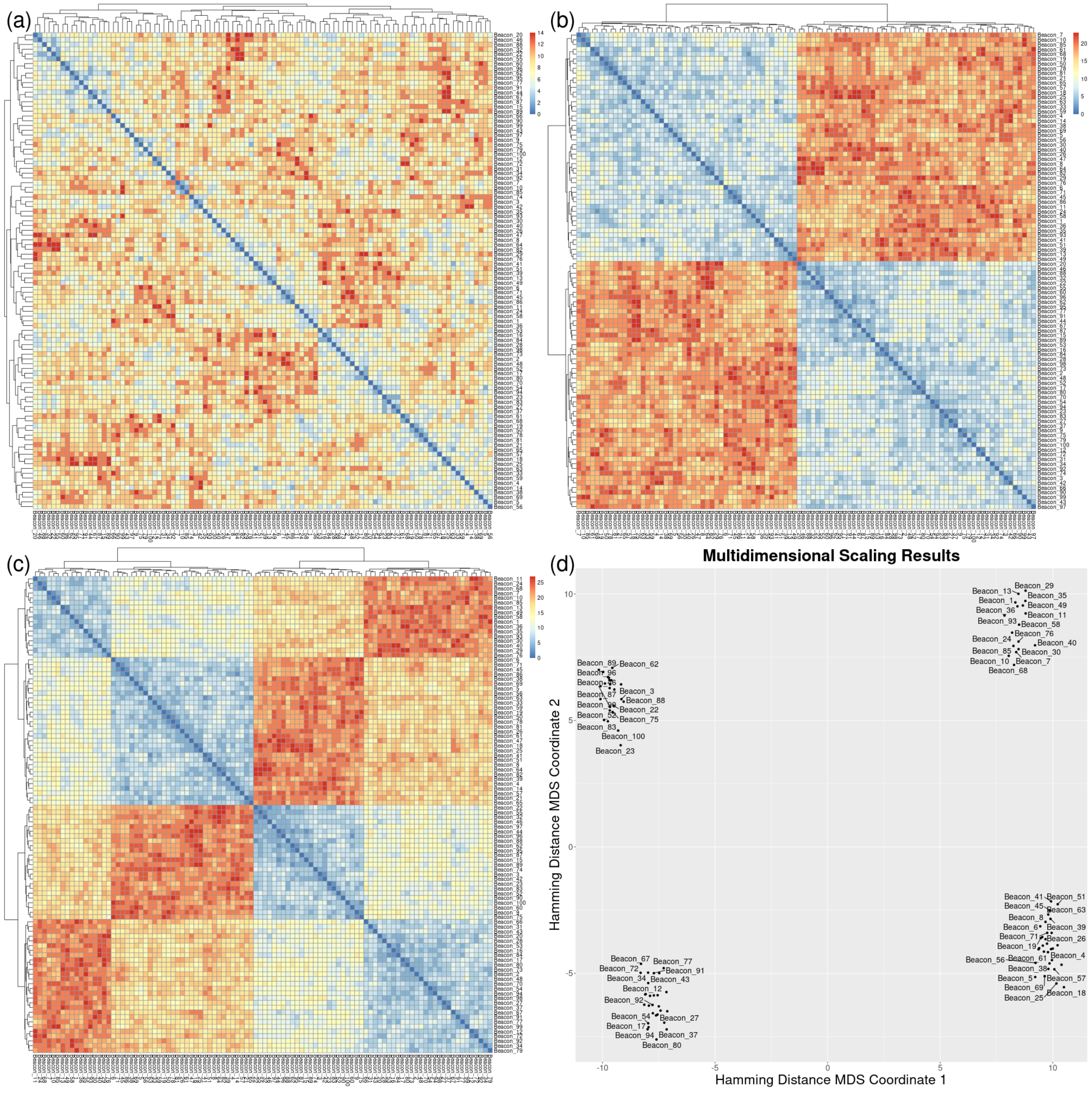

Figure SF5. Impact of assigning weights to variables in simulated data. Results from simulated data from the *individuals* entity of the Beacon v2 Models using Pheno-Ranker, created from the pairwise Hamming distance between all individuals. This simulation includes a cohort of 100 individuals, each with 2 *phenotypicFeatures*, 2 *diseases*, and 2 *treatments* (selected from a pool of 5), as well as *sex* and *ethnicity*. The objective of this

experiment was to demonstrate the effectiveness of assigning weights to variables. (a) Displays results including phenotypic measures, diseases, treatments and sex. (b) Shows outcomes when a weights file is applied, assigning a weight of 10 to the term 'Caucasian'. (c) Further includes the treatment 'RxNorm:1000000' (Tribenzor) with a weight of 5. (d) Illustrates multidimensional data scaling based on the data from (c). Figure results can be reproduced by using the commands at https://github.com/CNAG-Biomedical-Informatics/pheno-ranker/tree/main/share/fig.

Supporting Figure 6

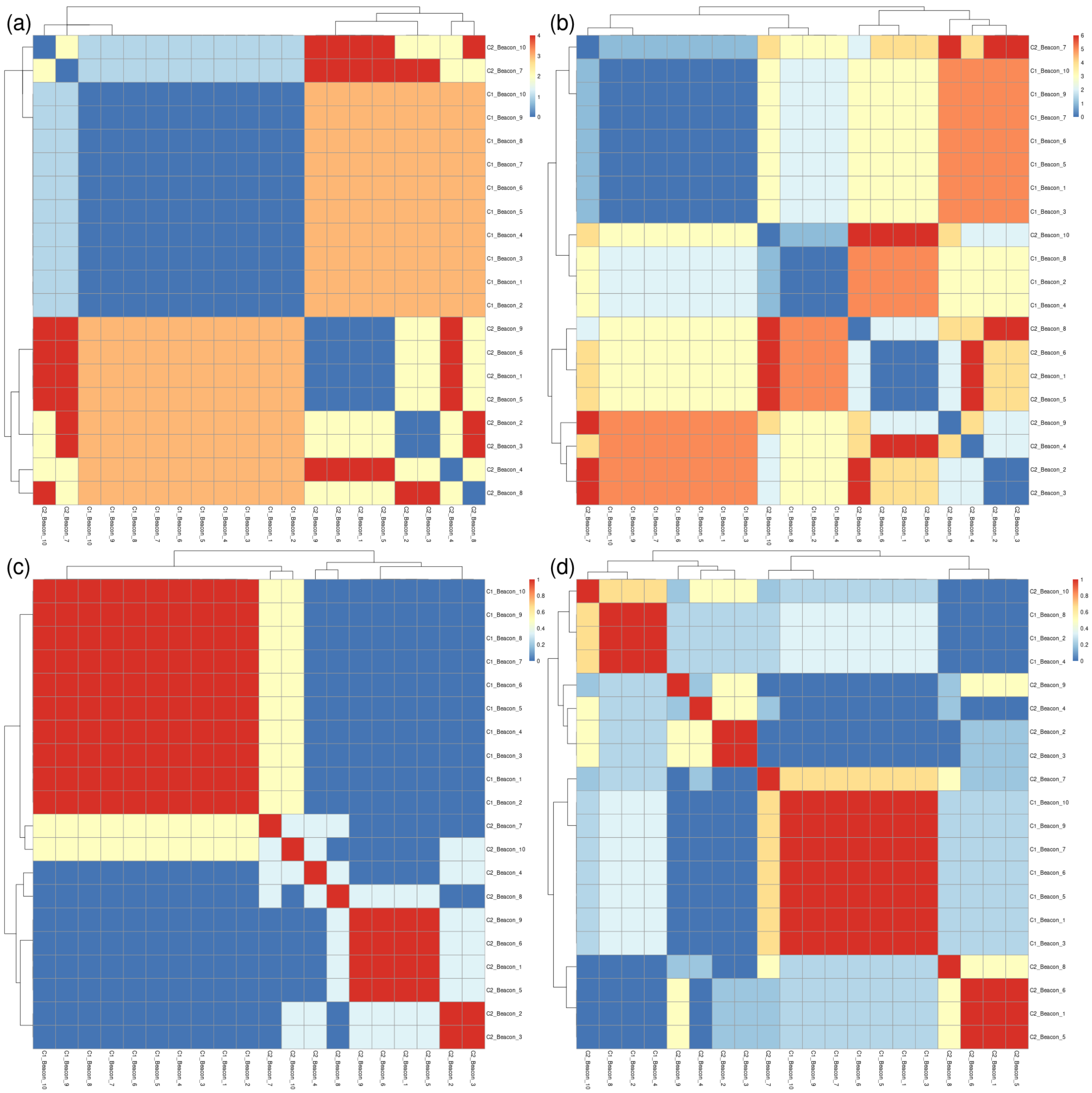

Figure SF6. Results with incomplete simulated data. Heatmaps and clusters from simulated Beacon v2 data (*individuals* entity) using Pheno-Ranker, showcasing two cohorts: C1, with 10 individuals each having 1 disease from a pool of 1, and C2, consisting of 10 individuals with 2 diseases from a pool of 5. The terms *sex* and *ethnicity* were assigned randomly. This demonstrates Pheno-Ranker's ability to capture similarities,

particularly in scenarios with incomplete data. The top two plots (a) and (b) are based on Hamming distance, while the bottom two plots (c) and (d) utilize the Jaccard index. (a) and (c) show results incorporating only the term *diseases*, while (b) and (d) include both *diseases* and *sex*. Figure results can be reproduced by using the commands at https://github.com/CNAG-Biomedical-Informatics/pheno-ranker/tree/main/share/fig.

Supporting Figure 7

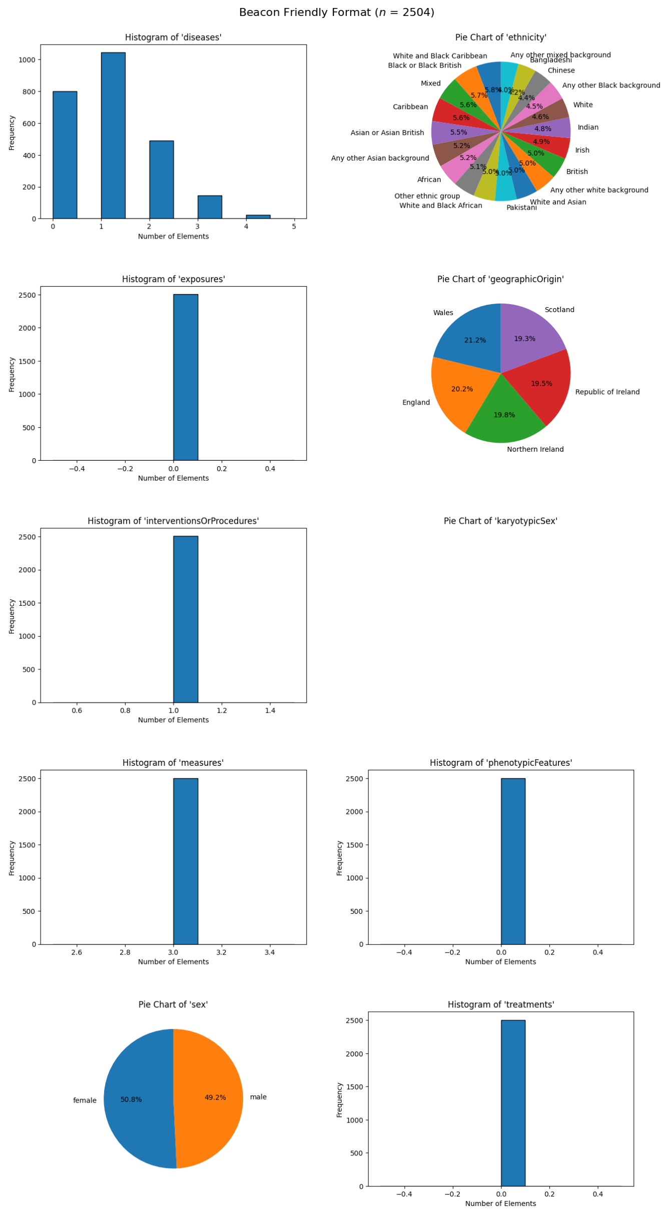

Figure SF7. Visualization from the *bff-pxf-plot* utility using synthetic cohort data. Image generated with the *bff-pxf-plot* utility in Pheno-Ranker's containerized version, showcasing data from the 'CINECA_synthetic_cohort_EUROPE_UK1' cohort. Figure results can be reproduced by using the commands at https://github.com/CNAG-Biomedical-Informatics/pheno-ranker/tree/main/share/fig.

Supporting Figure 8

Pheno-Ranker

ID: 107:week_0_arm_1

Date: 2023-12-07
Data type: BFF
This is an auto-generated report by Pheno-Ranker

diseases

[Item:0] diseaseCode_id
NCIT:C3138

ethnicity

id
NCIT:C41261

exposures

[Item:0] exposureCode_id
NCIT:C154329

[Item:0] unit_id
NCIT:C65108

[Item:1] exposureCode_id
NCIT:C2190

Figure SF8. Proof-of-concept report from patient data encoded as a QR code. PDF report generated from a QR code image, created using Pheno-Ranker's QR code utilities. The original QR code encapsulates patient information encoded as a binary vector. A detailed description of the process is available at https://cnag-biomedical-informatics.github.io/pheno-ranker/qr-code-generator/.

Supporting Figure 9

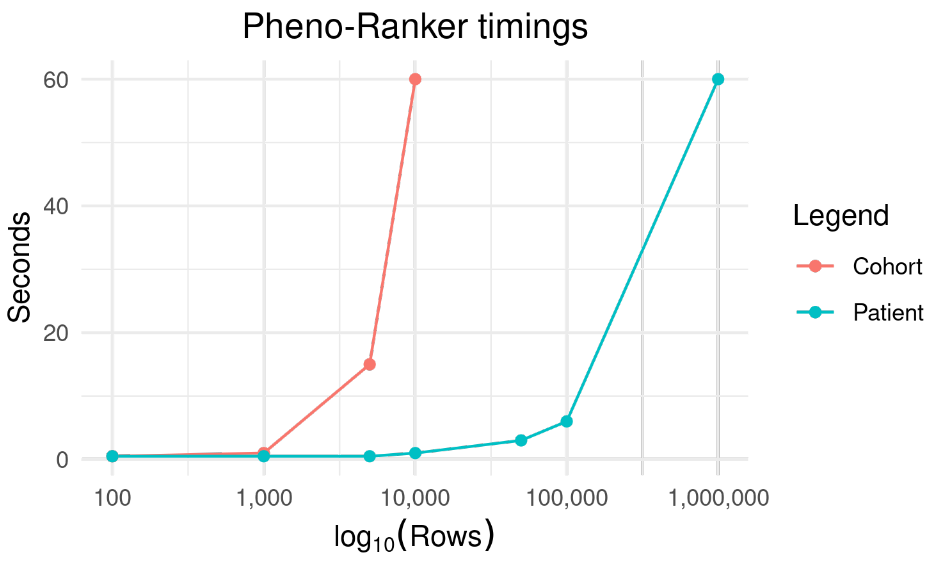

Figure SF9. Pheno-Ranker timing results for cohort and patient modes. Timings for Pheno-Ranker's command-line interface in *cohort* and *patient* modes using a dataset (CSV converted to JSON) with 19 variables (columns). Note: The x-axis is on a logarithmic scale. Tests were conducted on a single core of an Intel(R) Xeon(R) W-1350P @ 4.00GHz with 32GB RAM and SSD, on a Hewlett-Packard workstation.
