# Supplementary material for: Pheno-Ranker: a toolkit for comparison of phenotypic data stored in GA4GH standards and beyond
Source: BMC Bioinformatics. 2024 Dec 4;25:373. doi: 10.1186/s12859-024-05993-2 (PMC11616229; doi:10.1186/s12859-024-05993-2)
Supplement: Supplementary file 2 — Additional file2 (PDF 142 KB) [file 12859_2024_5993_MOESM2_ESM.pdf]

Supporting Table 1: Implementation challenges and solutions

**Command-line interface**	
**Faced challenges**	**Solution**
Handling nested JSON structures with arrays	When flattening complex JSON structures with arrays, elements are preceded by an index, which can interfere with the comparisons. For 1D arrays, we replace indexes with the label derived from the 'id' key of the required ontology term, as defined in the config file. For 2D arrays, we have implemented *ad hoc* parsing to support PXF term *interpretations*. If issues arise, we recommend addressing them by either: i) filtering out problematic variables using the config file, or ii) preprocessing the JSON data, such as converting arrays into objects. Any critical issues specific to BFF/PXF will be addressed by our team as they arise.
**Web App UI**	
**Faced challenges**	**Solution**
Single threaded nature of R rendered the Shiny application unresponsive when in use by many concurrent users	We resolved scaling issues by self-hosting ShinyProxy (shinyproxy.io), allowing us to serve one instance per user.
Strict Content-Security-Policy (CSP) headers break ShinyProxy	As suggested by the ShinyProxy developers we have removed `require-trusted-types-for 'script';` in our nginx configuration (https://github.com/openanalytics/shinyproxy/issues/476)
Resource intensive calculations	The Pheno-Ranker Perl module has been extensively optimized to minimize CPU and RAM consumption.<br><br>However, clustering or dimensionality reduction with R can still be quite CPU/RAM intensive, depending on the cohort size. Therefore, we have implemented a cutoff for the maximum number of individuals, currently set at 1000. This limit will be adjusted based on the usage of the playground and our available resources.

GDPR compliant login	To ensure full GDPR compliance, we aim to store minimal user information on our servers. Therefore, we use European Life Science Research Infrastructures (LS-RI) as our identity provider, allowing users to log in with their ORCID iD.
Maintenance complexity	It is best practice to split large and complex R Shiny applications into reusable modules. This approach reduces code duplication and enhances the maintainability of the application. For modularization we resorted to golem (Fay, Guyader, Rochette, et al. 2023).

Supporting Table 2: Overview of Pheno-Ranker Apps and utilities used

Software Name	Version / Language	Type	Short Description	Install Methods	Docs Ref
pheno-ranker	0.12 / Perl 5	CLI	Script to compare cohort and patient data	D, G, C	1
**Pheno-Ranker Web App**	0.0.0.9022 / R, JS	R Shiny App	Web app user interface to *pheno-ranker* CLI	D	2
Utils: ***bff-pxf-plot***	0.12 / Python 3	CLI	Script to plot summary statistics on BFF/PXF files	D, G	3
Utils: ***bff-pxf-simulator***	0.12 / Perl 5	CLI	Script to generate simulated data in BFF/PXF format	D, G, C	4
Utils: ***csv2pheno-ranker***	0.12 / Perl 5	CLI	Script to convert any CSV to JSON compatible with *pheno-ranker* CLI	D, G, C	5
Utils: ***pheno-ranker2barcode***, ***barcode2pheno-ranker*** and ***pheno-ranker2pdf***	0.12 / Python 3	CLI	Script to convert P-R vector to QR codes, script to convert back QR codes to P-R and script to convert vector to PDF report	D, G	6

Install Methods: D = Docker, G = GitHub, C = CPAN
Documentation References:

1. pheno-ranker Docs
2. Pheno-Ranker Web App Docs
3. bff-pxf-plot Docs
4. bff-pxf-simulator Docs
5. csv2pheno-ranker Docs
6. QR Code Tools Docs
