# Supplementary material for: Pheno-Ranker: a toolkit for comparison of phenotypic data stored in GA4GH standards and beyond
Source: BMC Bioinformatics. 2024 Dec 4;25:373. doi: 10.1186/s12859-024-05993-2 (PMC11616229; doi:10.1186/s12859-024-05993-2)
Supplement: Supplementary file 3 — Additional file3 (PDF 62 KB) [file 12859_2024_5993_MOESM3_ESM.pdf]

Statistical assessment of the metrics

In addition to the similarity metrics, we assess the statistical significance by calculating Z-scores and p-values for both observed (empirical) and estimated values.

The Z-score is determined as:

$$Z_{score} = \frac{x - \mu}{\sigma}$$

where x represents the specific metric value for a given patient, μ is the mean of the patient-cohort values, and σ denotes the standard deviation of these values. A negative Z-score in our analysis is indicative of a favorable outcome. The estimated Z-score for the Hamming distance is calculated from binary strings having an alignment length equal to the number of '1's in the two vectors, on which each position has a 50% chance of being a mismatch, independent of other positions. See formulas and additional information on the online documentation at https://cnag-biomedical-informatics.github.io/pheno-ranker/patient.

We determined the statistical significance (p-value) of our findings by applying the cumulative distribution function (CDF) to these Z-scores, utilizing one-tailed (left-tailed) hypothesis testing. By comparing the observed and estimated Z-scores, users can assess the statistical significance of the similarity between the patient and the individuals in the cohort in a more comprehensive manner. Together, these measures address potential biases introduced by the cohort size and the length of the binary digit strings, leading to a more robust evaluation of matches and aiding decision-making in data analysis. However, if the comparison involves only a few variables, the provided statistics may be overly stringent and should be interpreted as a guide.
