# Supplementary material for: Pheno-Ranker: a toolkit for comparison of phenotypic data stored in GA4GH standards and beyond
Source: BMC Bioinformatics. 2024 Dec 4;25:373. doi: 10.1186/s12859-024-05993-2 (PMC11616229; doi:10.1186/s12859-024-05993-2)
Supplement: Supplementary file 4 — Additional file4 (PDF 145 KB) [file 12859_2024_5993_MOESM4_ESM.pdf]

Part A: Detailed Listing of PRECISESADS Clinical Consortium members

Lorenzo Beretta[1], Barbara Vigone[1],
Jacques-Olivier Pers[2], Alain Saraux[2], Valérie Devauchelle-Pensec[2], Divi Cornec[2], Sandrine Jousse-Joulin [2],
Bernard Lauwerys[3], Julie Ducreux[3], Anne-Lise Maudoux[3],
Carlos Vasconcelos[4], Ana Tavares[4], Esmeralda Neves[4], Raquel Faria [4], Mariana Brandão[4],
Ana Campar[4], António Marinho[4], Fátima Farinha[4], Isabel Almeida[4]
Miguel Angel Gonzalez-Gay[5], Ricardo Blanco Alonso[5], Alfonso Corrales Martínez[5],
Ricard Cervera[6], Ignasi Rodríguez-Pintó[6], Gerard Espinosa [6],
Rik Lories[7], Ellen De Langhe[7],
Nicolas Hunzelmann[8], Doreen Belz[8],
Torsten Witte[9], Niklas Baerlecken [9],
Georg Stummvoll[10], Michael Zauner[10], Michaela Lehner [10],
Eduardo Collantes[11], Rafaela Ortega-Castro[11], Mª Angeles Aguirre-Zamorano[11], Alejandro Escudero-Contreras[11], Mª Carmen Castro-Villegas [11], Yolanda Jiménez Gómez[11],
Norberto Ortego[12], María Concepción Fernández Roldán [12],
Enrique Raya[13], Inmaculada Jiménez Moleón [13],
Enrique de Ramon[14], Isabel Díaz Quintero[14],
Pier Luigi Meroni[15], Maria Gerosa[15], Tommaso Schioppo[15], Carolina Artusi[15],
Carlo Chizzolini[16], Aleksandra Dufour[16], Donatienne Wynar[16],
Laszló Kovács[17], Attila Balog[17], Magdolna Deák[17], Márta Bocskai[17], Sonja Dulic[17], Gabriella Kádár[17],
Falk Hiepe[18], Velia Gerl[18], Silvia Thiel[18],
Manuel Rodriguez Maresca[19], Antonio López-Berrio[19], Rocío Aguilar-Quesada[19], Héctor Navarro-Linares [19]
Yiannis Ioannou[20], Chris Chamberlain[20], Jacqueline Marovac[20],
Marta Alarcón Riquelme[21], Tania Gomes Anjos[21].

Funding: This work has received support from the EU/EFPIA Innovative Medicines Initiative Joint Undertaking (PRECISESADS, grant n. 115565) including in-kind contributions from the EFPIA members involved.

www.precisesads.eu

Affiliations

1 Referral Center for Systemic Autoimmune Diseases, Fondazione IRCCS Ca' Granda Ospedale Maggiore Policlinico di Milano, Italy.
2 Centre Hospitalier Universitaire de Brest, Hospital de la Cavale Blanche, Brest, France.
3 Pôle de pathologies rhumatismales systémiques et inflammatoires, Institut de Recherche Expérimentale et Clinique, Université catholique de Louvain, Brussels, Belgium.
4 Centro Hospitalar do Porto, Portugal.
5 Hospital Universitario Marqués de Valdecilla, IDIVAL, Universidad de Cantabria, Santander, Spain.
6 Hospital Clinic I Provicia, Institut d'Investigacions Biomèdiques August Pi i Sunyer, Barcelona, Spain.
7 Katholieke Universiteit Leuven, Belgium.
8 Klinikum der Universitaet zu Koeln, Cologne, Germany.
9 Medizinische Hochschule Hannover, Germany.
10 Medical University Vienna, Vienna, Austria.
11 Servicio Andaluz de Salud, Hospital Universitario Reina Sofía Córdoba, Spain.
12 Servicio Andaluz de Salud, Complejo hospitalario Universitario de Granada (Hospital Universitario San Cecilio), Spain.
13 Servicio Andaluz de Salud, Complejo hospitalario Universitario de Granada (Hospital Virgen de las Nieves), Spain.
14 Servicio Andaluz de Salud, Hospital Regional Universitario de Málaga, Spain
15 Università degli studi di Milano, Milan, Italy.
16 Hospitaux Universitaires de Genève, Switzerland.
17 University of Szeged, Szeged, Hungary.
18 Charite, Berlin, Germany.
19 Andalusian Public Health System Biobank, Granada, Spain
20 UCB Pharma, Slough, United Kingdom (PRECISESADS Project office)
21 Department of Medical Genomics, Center for Genomics and Oncological Research (GENYO), Granada, Spain (PRECISESADS Project Office)

Part B: Names of PRECISESADS Ethics committees and associated members

Lorenzo Beretta[1], Barbara Vigone[21],
Jacques-Olivier Pers2, Alain Saraux2, Valérie Devauchelle-Pensec2, Divi Cornec2, Sandrine Jousse-Joulin [21],
Bernard Lauwerys[3], Julie Ducreux[3], Anne-Lise Maudoux[21],
Carlos Vasconcelos[4], Ana Tavares[4], Esmeralda Neves[4], Raquel Faria [21], Mariana Brandão[4],
Ana Campar[4], António Marinho[4], Fátima Farinha[4], Isabel Almeida[4]
Miguel Angel Gonzalez-Gay Mantecón5, Ricardo Blanco Alonso5, Alfonso Corrales Martínez[21],
Ricard Cervera[6], Ignasi Rodríguez-Pintó[6], Gerard Espinosa [21],
Rik Lories[7], Ellen De Langhe[21],
Nicolas Hunzelmann[8], Doreen Belz[21],
Torsten Witte[9], Niklas Baerlecken [21],
Georg Stummvoll[10], Michael Zauner[10], Michaela Lehner [21],
Eduardo Collantes[11], Rafaela Ortega-Castro[11], Mª Angeles Aguirre-Zamorano[11], Alejandro Escudero-Contreras[11], Mª Carmen Castro-Villegas [21]
Norberto Ortego[12], María Concepción Fernández Roldán [21],
Enrique Raya[13], Inmaculada Jiménez Moleón [21],
Enrique de Ramon[14], Isabel Díaz Quintero[21],
Pier Luigi Meroni[15], Maria Gerosa[15], Tommaso Schioppo[15], Carolina Artusi[21],
Carlo Chizzolini[16], Aleksandra Zuber[16], Donatienne Wynar[21],
Laszló Kovács[17], Attila Balog[17], Magdolna Deák[17], Márta Bocskai[17], Sonja Dulic[17], Gabriella Kádár[21],
Falk Hiepe[18], Velia Gerl[18], Silvia Thiel[21],
Manuel Rodriguez Maresca[19], Antonio López-Berrio[19], Rocío Aguilar-Quesada[19], Héctor Navarro-Linares [21]

Name of the committee (affiliation)

---

1 Referral Center for Systemic Autoimmune Diseases, Fondazione IRCCS Ca' Granda Ospedale Maggiore Policlinico di Milano, Comitato Etico Italy.
2 Centre Hospitalier Universitaire de Brest, Hospital de la Cavale Blanche, Avenue Tanguy Prigent 29609, Brest, France. Comite de Protection des Personnes Ouest VI
3 Pôle de pathologies rhumatismales systémiques et inflammatoires, Institut de Recherche Expérimentale et Clinique, Université catholique de Louvain, Brussels, Belgium. Comité d`Èthique Hospitalo-Facultaire
4 Centro Hospitalar do Porto, Portugal. Comissao de ética para a Saude – CES do CHP
5 Servicio Cantabro de Salud, Hospital Universitario Marqués de Valdecilla, Santander, Spain. Comite ético de investigacion clinical de Cantabria. IDIVAL
6 Hospital Clinic I Provicia, Institut d'Investigacions Biomèdiques August Pi i Sunyer, Barcelona, Spain. Comité Ética de Investigación Clínica del Hospital Clínic de Barcelona. Hospital Clínic de Barcelona
7 Katholieke Universiteit Leuven, Belgium. Commissie Medische Ethiek UZ KU Leuven /Onderzoek
8 Klinikum der Universitaet zu Koeln, Cologne, Germany. Geschaftsstelle Ethikkommission
9 Medizinische Hochschule Hannover, Germany. Ethikkommission
10 Medical University Vienna, Vienna, Austria. Ethik Kommission. Borschkegasse.
11 Servicio Andaluz de Salud, Hospital Universitario Reina Sofía Córdoba, Spain. Comité de Ética e la Investigación de Centro de Granada (CEI – Granada)
12 Servicio Andaluz de Salud, Complejo hospitalario Universitario de Granada (Hospital Universitario San Cecilio), Spain. Comité de Ética e la Investigación de Centro de Granada (CEI – Granada)
13 Servicio Andaluz de Salud, Complejo hospitalario Universitario de Granada (Hospital Virgen de las Nieves), Spain. Comité de Ética e la Investigación de Centro de Granada (CEI – Granada)
14 Servicio Andaluz de Salud, Hospital Regional Universitario de Málaga, Spain. Comité de Ética e la Investigación de Centro de Granada (CEI – Granada)
15 Università degli studi di Milano, Milan, Italy. Policlinico di Milano, Comitato Etico Italy.
16 Hospitaux Universitaires de Genève, Switzerland. DEAS –Commission Cantonale d`´ethique de la recherche Hopitaux universitaires de Geneve
17 University of Szeged, Szeged, Hungary. Csongrad Megyei Kormanyhivatal
18 Charite, Berlin, Germany. Ethikkommission.
19 Andalusian Public Health System Biobank, Granada, Spain.
