# Supplementary material for: Pheno-Ranker: a toolkit for comparison of phenotypic data stored in GA4GH standards and beyond
Source: BMC Bioinformatics. 2024 Dec 4;25:373. doi: 10.1186/s12859-024-05993-2 (PMC11616229; doi:10.1186/s12859-024-05993-2)
Supplement: Supplementary file 6 — Additional file6 (PDF 224 KB) [file 12859_2024_5993_MOESM6_ESM.pdf]

Addendum: Proposal for Implementing Pheno-Ranker in a Federated Network

<u>Use Case A: Inter-Hospital Network</u>

The current version of Pheno-Ranker is designed for file-based operations and initiates calculations from scratch each time. To adapt the algorithm for use in multiple hospitals without directly sharing clinical data, we propose the following approach:

1. Preparation Stage:
<u>Vector Standardization</u>: Ensure all hospitals use a standardized vector format.

a. Store each patient's vector in a local-database.
   - `“id_1": "1101010101010...n",`
   - `"id_2": "0101010101000...n`
b. Utilize a network aggregator to regularly update a global reference vector. Each update gets a new version identifier.
c. Periodically update the vector database at each site to ensure current data.

<u>Privacy Protocols</u>: Set up differential privacy mechanisms or encryption protocols.
<u>Threshold Agreement:</u> Establish a common threshold for the Hamming distance (or other metric) for matches.

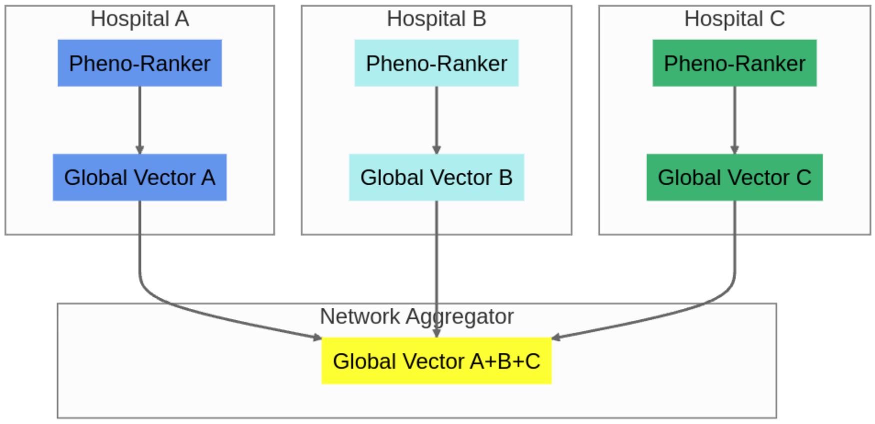

2. Query Initiation:
The querying hospital prepares a vector representation of the individual or set of individuals.
The vector is processed using the agreed-upon privacy protocols.

3. Aggregator Mediation:
The querying hospital sends the processed vector to the network aggregator.
The network aggregator distributes the query to all hospitals in the federated network.

4. Local Computation:

Each receiving hospital computes the Hamming distance against its local patient vectors. The computation is done entirely within the local environment of each hospital.

5. Thresholding:
Each hospital applies the agreed-upon thresholding to identify vectors that are considered a "match."

6. Response to Aggregator:
Each hospital sends its response (list of matching vectors, counts, etc.) back to the network aggregator.

7. Aggregation:
The network aggregator collects all the responses, processes them, and sends the aggregated result to the querying hospital.

8. Post-Processing:
The querying hospital undertakes further analysis, potentially reaching out to specific hospitals for more information based on the aggregated results and decides on subsequent actions.

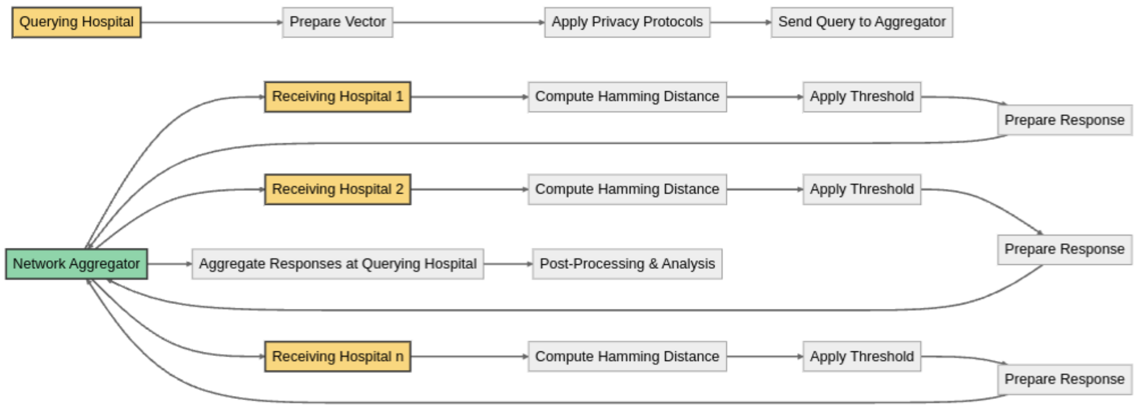

<u>Use Case B: Beacon v2 Network:</u>

Currently, the `/individuals` endpoint from the Beacon v2 API can be queried, and, as long as the user has appropriate access to the record-level data, the response can be parsed and saved as a text file (i.e., BFF). Pheno-Ranker can then be executed locally, either via the command-line interface (CLI) or through the Web App UI. While this approach works, it becomes cumbersome when the goal is to search for similar patients across multiple Beacon instances.

To facilitate Pheno-Ranker's integration into the Beacon v2 API ecosystem, we propose two distinct pathways for query submission to enhance flexibility and security (both requiring `POST` requests):

i) The first method mirrors the approach used in hospital networks, where queries leverage a precomputed vector. This ensures secure and efficient similarity evaluations against an existing database. To support this, a Beacon aggregator would periodically gather ontology terms via the *filtering_terms* endpoint from each Beacon v2 API, thereby creating a global lookup table.

ii) Alternatively, centers may submit queries using actual JSON data (either BFF or PXF objects), which should be anonymized or meet the network's security standards. This option allows the recipient site to perform similarity analyses either on their precomputed data or on-the-fly using Pheno-Ranker's CLI or module, offering greater adaptability.

The response schema can either adhere to the Beacon v2 specification or be adapted to include similarity metrics, enhancing the utility and adaptability of the integration.

<u>Draft Proposal for the JSON Schema of the 'phenoRanker' Query Parameter (in YAML format; subject to future modifications to align with Beacon v2 specifications):</u>

```yaml
$schema: "https://json-schema.org/draft/2020-12/schema"
type: object
properties:
  meta:
    type: object
    properties:
      apiVersion:
        type: string
        description: "The version of the Beacon API being used."
        enum: ["2.0", "2.1"]
    required:
      - apiVersion
  query:
    type: object
    properties:
      requestParameters:
        type: object
        properties:
          phenoRanker:
            type: array
            description: "An array of phenoRanker objects, all of the same type
('vector', 'bff', or 'pxf')."
            items:
              type: object
              properties:
                info:
                  type: string
                  description: "Optional additional information about the
phenoRanker object."
              oneOf:
                - properties:
                    vector:
                      type: string
                      pattern: "^[01]+$"
```

```
           description: "A binary string representing a vector."
           example: "1010101"
          version:
           type: string
           description: "Version of the vector data."
           example: "1.0.0"
         required:
          - vector
          - version
        - properties:
          bff:
           type: object
           description: "An object representing BFF data."
          version:
           type: string
           description: "Version of the BFF data."
           example: "2.0"
           enum: ["2.0"]
         required:
          - bff
          - version
        - properties:
          pxf:
           type: object
           description: "An object representing PXF data."
          version:
           type: string
           description: "Version of the PXF data."
           example: "2.0"
           enum: ["2.0"]
         required:
          - pxf
          - version
       description: "Each item must contain exactly one of 'vector',
'bff', or 'pxf'."
      minItems: 1
      additionalItems: false
     # Include other requestParameters as needed
    required:
     - phenoRanker
   # Include 'filters' or 'pagination' if necessary
  required:
   - requestParameters
required:
 - meta
 - query
description: "Schema for a Beacon v2 API query incorporating phenoRanker data."
```

<u>Example Queries:</u>

1. Example with 'vector' items

```
{
 "meta": {
  "apiVersion": "2.0"
```

```
    },
    "query": {
      "requestParameters": {
        "phenoRanker": [
          {
            "vector": "1010101",
            "version": "1.0.0",
            "info": "Sample vector data"
          },
          {
            "vector": "1110001",
            "version": "1.0.0",
            "info": "Another vector data"
          }
        ]
      }
    }
  }
```

2. Example with ‘bff’ items

```
{
  "meta": {
    "apiVersion": "2.0"
  },
  "query": {
    "requestParameters": {
      "phenoRanker": [
        {
          "bff": { "someKey": "someValue1" },
          "version": "2.0"
        },
        {
          "bff": { "someKey": "someValue2" },
          "version": "2.0"
        }
      ]
    }
  }
}
```
